# A Macrophage Response to *Mycobacterium leprae* Phenolic Glycolipid Initiates Nerve Damage in Leprosy

**DOI:** 10.1016/j.cell.2017.07.030

**Published:** 2017-08-24

**Authors:** Cressida A. Madigan, C.J. Cambier, Kindra M. Kelly-Scumpia, Philip O. Scumpia, Tan-Yun Cheng, Joseph Zailaa, Barry R. Bloom, D. Branch Moody, Stephen T. Smale, Alvaro Sagasti, Robert L. Modlin, Lalita Ramakrishnan

**Affiliations:** 1 Division of Dermatology, Department of Medicine, David Geffen School of Medicine, University of California, Los Angeles, Los Angeles, CA 90095, USA; 2 Department of Microbiology, Immunology, and Molecular Genetics, David Geffen School of Medicine, University of California, Los Angeles, Los Angeles, CA 90095, USA; 3 Department of Microbiology, University of Washington, Seattle, WA 98195, USA; 4 Department of Immunology, University of Washington, Seattle, WA 98195, USA; 5 Division of Rheumatology, Immunology, and Allergy, Brigham and Women’s Hospital, Harvard Medical School, Boston, MA 02115, USA; 6 Harvard School of Public Health, Boston, MA 02115, USA; 7 Department of Molecular, Cell, and Developmental Biology, University of California, Los Angeles, Los Angeles, CA 90095, USA; 8 Department of Medicine, University of Washington, Seattle, WA 98195, USA; 9 MRC Laboratory of Molecular Biology, Molecular Immunity Unit, Department of Medicine, University of Cambridge, Cambridge CB2 OQH, UK; 10 Molecular Biology Institute, University of California, Los Angeles, Los Angeles, CA 90095, USA

**Keywords:** mycobacteria, macrophage, leprosy, zebrafish, phenolic glycolipid, nerve damage, myelin

## Abstract

*Mycobacterium leprae* causes leprosy and is unique among mycobacterial diseases in producing peripheral neuropathy. This debilitating morbidity is attributed to axon demyelination resulting from direct interaction of the *M. leprae-*specific phenolic glycolipid 1 (PGL-1) with myelinating glia and their subsequent infection. Here, we use transparent zebrafish larvae to visualize the earliest events of *M. leprae*-induced nerve damage. We find that demyelination and axonal damage are not directly initiated by *M. leprae* but by infected macrophages that patrol axons; demyelination occurs in areas of intimate contact. PGL-1 confers this neurotoxic response on macrophages: macrophages infected with *M. marinum*-expressing PGL-1 also damage axons. PGL-1 induces nitric oxide synthase in infected macrophages, and the resultant increase in reactive nitrogen species damages axons by injuring their mitochondria and inducing demyelination. Our findings implicate the response of innate macrophages to *M. leprae* PGL-1 in initiating nerve damage in leprosy.

## Highlights

• *Mycobacterium leprae* infection of zebrafish damages nerves, causing demyelination
• Nerve damage requires *M. leprae* phenolic glycolipid (PGL-1) and host macrophages
• PGL-1 induces macrophages to produce excess nitric oxide
• Excess nitric oxide damages nerves by damaging their mitochondria

## Graphical Abstract

**Figure:**
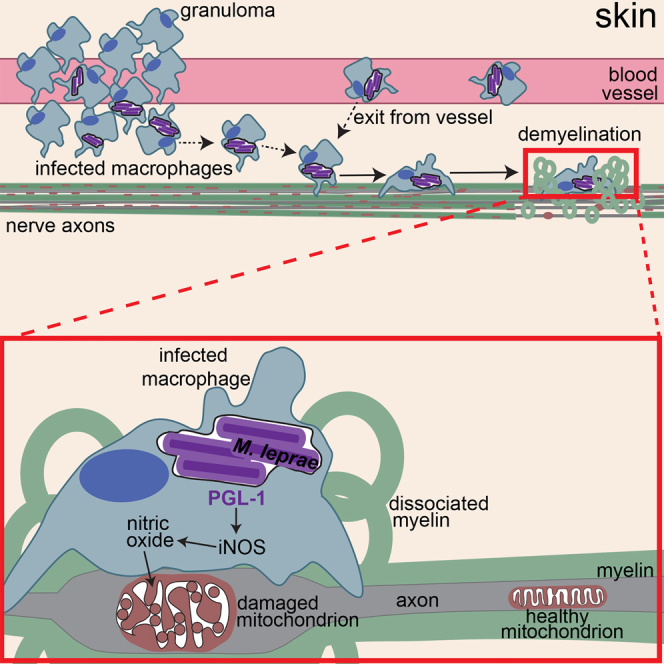

## Teaser

A new model system for leprosy enables the discovery that macrophages, and not the causative bacterium *M. leprae*, initiate host nerve demyelination and axonal damage.

## Introduction

Leprosy, like tuberculosis, presents as a granulomatous disease. These granulomas are usually cutaneous, reflecting the ∼30°C growth optimum of *M. leprae*, similar to that of the human skin (∼34°C) (Bierman, 1936, Renault and Ernst, 2015, Truman and Krahenbuhl, 2001*). M. leprae* is the only mycobacterial infection that causes widespread demyelinating neuropathy, which results in the main morbidities of leprosy, including autoamputation of digits and blindness (Renault and Ernst, 2015). Understanding the pathogenesis of leprosy neuropathy has been stymied by the inability to culture *M. leprae*, which has undergone severe reductive evolution of its genome to become an obligate intracellular pathogen (Cole et al., 2001, Scollard et al., 2006). The lack of genetic tools for studying *M. leprae* is compounded by the lack of genetically tractable animal models that mimic the human disease. The athymic mouse footpad is used to grow *M. leprae* for research purposes, but mice do not manifest neurological disease (Scollard et al., 2006). While the nine-banded armadillo develops neuropathy following infection with *M. leprae*, it suffers from a paucity of molecular and genetic tools (Truman et al., 2014). Consequently, our understanding of the pathogenesis of leprosy neuropathy in vivo largely comes from studies of patients; however, the 4- to 10-year delay in the onset of symptoms largely precludes studies of the early events that lead to neuropathy (Noordeen, 1994).

Leprosy can present as a clinical spectrum; at the poles of this spectrum are paucibacillary (or tuberculoid) and multibacillary (or lepromatous) disease. The former is characterized by a vigorous immune response, while the latter, an ineffective one (Scollard et al., 2006). Neuropathy features prominently in both forms of the disease. Hence, bacterial determinants and host immune responses likely play roles in leprosy neuropathy, although the relative importance and mechanisms by which each contributes to nerve injury are poorly understood. In vitro studies suggest a model wherein *M. leprae* directly causes demyelination by infecting and dedifferentiating the Schwann cells that myelinate peripheral nerves (Rambukkana et al., 2002, Truman et al., 2014). These studies identified an *M. leprae* outer membrane lipid, phenolic glycolipid 1 (PGL-1), that is critical for binding to laminin α2, an interaction thought to promote infection of the Schwann cells (Ng et al., 2000). However, this model fails to explain the neuropathy in paucibacillary leprosy, in which bacteria are seldom observed within nerve lesions (Shetty and Antia, 1996). Rather, a pathogenic CD4 T cell response, possibly acting through secreted cytokines, is implicated in paucibacillary disease (Renault and Ernst, 2015). Further, the specific contributions of macrophages in leprosy neuropathy are unknown, although they are commonly infected and almost universally present in affected nerves (Job, 1973, Shetty and Antia, 1996).

The developing zebrafish is an effective model for studying mycobacterial pathogenesis using *M. marinum*, a close genetic relative of the *M. tuberculosis* complex and the agent of fish tuberculosis (Ramakrishnan, 2004). The genetic tractability of the zebrafish, coupled with the optical transparency of its larva, allows host-bacterium interactions to be monitored in real-time, providing critical insights into disease pathogenesis (Ramakrishnan, 2004). Furthermore, adaptive immunity is not yet present at the larval developmental stage, permitting study of host-pathogen interactions in the sole context of innate immunity (Davis et al., 2002). Here, we exploit the optical transparency of larval zebrafish to directly visualize the earliest interactions of *M. leprae* with macrophages (Davis et al., 2002), and the initial events in nerve injury (Czopka, 2016). We use *M. marinum* as a comparator for these studies because, like *M. leprae*, it grows at ∼30°C and produces cutaneous granulomatous infections in humans (Ramakrishnan, 2004). However, it does not cause neuropathy. Our studies reveal that *M. leprae* interacts with macrophages and incites granulomas similar to *M. marinum* (Madigan et al., 2017), but is unique in its ability to produce demyelination and axonal damage. We show that the innate macrophage response to PGL-1 triggers demyelination *in vivo*, even before bacilli have detectably infected the glia. Finally, we determine the mechanism of nerve damage using *M. leprae* and *M. marinum* engineered to synthesize PGL-1.

## Results

### *M. leprae* Elicits Typical Responses in Macrophages of Zebrafish Larvae

**Figure 1:**
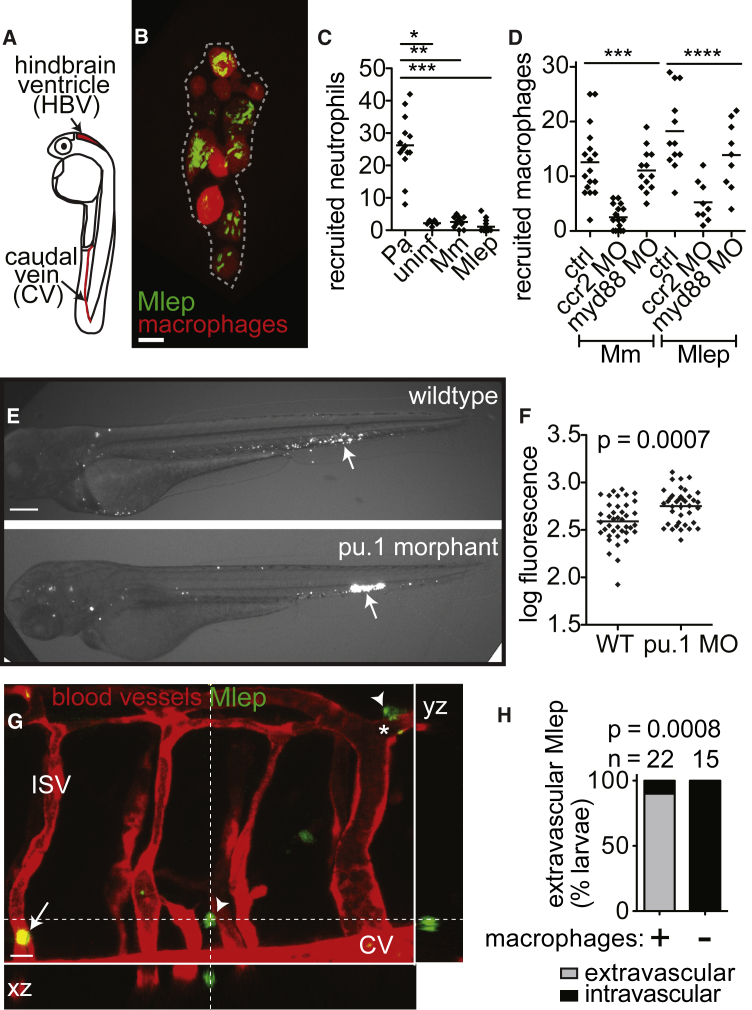
Early *M. leprae*-Macrophage Interactions Are Typical of Other Mycobacterial Infections (A) Diagram of larva 2 days post-fertilization (dpf) with injection sites indicated. (B) Representative confocal image of an early aggregate of fluorescent macrophages (dashed line) adjacent to the yolk sac in a 6 dpf *mpeg1:Brainbow* larva at 4 days post-infection (dpi) with ∼10^4^ fluorescent *M. leprae*. Scale bar, 10 μm. (C) Mean number of neutrophils recruited to the hindbrain ventricle after injection of ∼100 colony-forming units (CFUs) of *P. aeruginosa* (Pa), *M. marinum*, or *M. leprae* in a 2 dpf larva at 4 hr post-infection; ^∗^p < 0.05; ^∗∗^p < 0.01; ^∗∗∗^p < 0.001 (one-way ANOVA with Bonferroni’s post-test). (D) Mean number of macrophages recruited to *M. marinum* (Mm) or *M. leprae* (Mlep) injection, like in (C), in wild-type (ctrl) animals or those made deficient in CCR2 or MyD88 by morpholino (MO) injection; ^∗∗∗^p < 0.001, ^∗∗∗∗^p ≤ 0.0001 (one-way ANOVA with Bonferroni’s post-test). (E) Representative fluorescent images of 2 dpi (4 dpf) WT or macrophage-deficient pu.1 morphant larvae, infected with fluorescent *M. leprae* like in (B); the arrow indicates the injection site. Scale bar, 100 μm. (F) Mean bacterial burden of larvae in (E); unpaired Student’s t test. (G) Representative confocal image of the fluorescent vasculature of a 2 dpi (4 dpf) *kdrl:dsRed* larva infected with fluorescent *M. leprae*; bacteria reside within macrophages, apparent by Nomarski microscopy (Figure S1). The arrow indicates *M. leprae* retained within vessels; arrowheads indicate *M. leprae* outside of vessels; ISV, intersegmental vessel; asterisk, *M. leprae*-infected macrophage surrounding the abluminal surface of the vessel. (H) Proportion of larvae in (G) with *M. leprae* disseminated outside or contained within the vasculature, 4 days after caudal vein infection, in larvae depleted of macrophages, or not, by clondronate injection (Fisher’s exact test). n = number of larvae per group; all data representative of at least three separate experiments. See also Figure S1.

To determine if zebrafish larvae might be a useful model for studying early *M. leprae* infection, we first examined the earliest interactions of *M. leprae* with phagocytes, by injecting bacteria into the caudal vein or the hindbrain ventricle (Figure 1A), where phagocytes are rarely observed in the absence of infection (Davis et al., 2002). Aggregates of infected macrophages formed within 4 days (Figure 1B), similar to the case with *M. marinum* infection (Davis et al., 2002). Prior studies indicate that phagocyte recruitment to *M. marinum* infection is unique in two respects: (1) neutrophils are not recruited to the initial site of infection (Yang et al., 2012), and (2) macrophage recruitment is independent of TLR-signaling, but dependent on the monocyte chemokine CCL2 and its receptor CCR2 (Cambier et al., 2014). *M. leprae* shared both of these features with *M. marinum*: neutrophils were not recruited, whereas macrophages were (Figures 1C and 1D). Further, this recruitment was TLR/MyD88 independent and CCL2/CCR2 dependent (Figure 1D) (Cambier et al., 2014). The *M. marinum* phenolic glycolipid (PGL*-mar*) induces CCL2 expression and mediates CCL2/CCR2-dependent macrophage recruitment (Cambier et al., 2014, Cambier et al., 2017), suggesting that PGL-1 may play a similar role in *M. leprae* infection.

**Figure S1:**
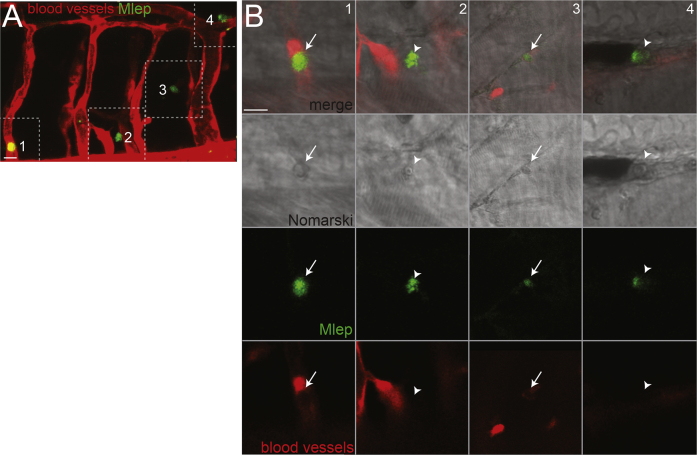
*M. leprae*-Infected Macrophages Escape Circulation, Related to Figures 1G and 1H (A) Confocal image from Figure 1G of a 4 dpf *kdrl:dsRed* larva, which has fluorescent blood vessels, 2 days post-infection (dpi) with fluorescent *M. leprae*; dashed lines define insets (1-4) shown in B. 10 μm bar. (B) Monochannel and merged images of insets from A, showing *M. leprae* within cells, likely macrophages, which are visible by Nomarski microscopy. Arrows, intracellular *M. leprae* retained in vessels; arrowheads, intracellular *M. leprae* outside vessels. 10 μm bar.

Macrophages play a dichotomous role in controlling *M. marinum* infection: they restrict bacterial numbers, while promoting dissemination of bacteria from the infection site into deeper tissues (Clay et al., 2007). Similar to the case observed for *M. marinum*, *M. leprae*-infected animals depleted of macrophages using the *pu.1* morpholino (Clay et al., 2007) displayed higher bacterial burdens (Figures 1E and 1F). The increased bacterial burden in the *pu.1* morphants is likely due to the lack of bacterial killing, rather than bacterial replication. The doubling time of *M. leprae* is approximately 12 days (Levy and Ji, 2006); therefore, most bacteria would not have replicated in the larvae during the 2-day infection. In addition, we assessed the role of macrophages in *M. leprae* dissemination, by infecting animals with fluorescent vascular endothelial cells (*kdrl:dsRed).* By 2 days post-infection (dpi) (4 dpf), *M. leprae* escaped the vasculature and entered peripheral tissues in the majority of wild-type, but not macrophage-depleted, larvae (Figures 1G and 1H). Furthermore, in wild-type animals, *M. leprae* resided in macrophages (apparent by Nomarski imaging in Figure S1), suggesting these cells carried *M. leprae* from the circulation into tissues. This is reminiscent of zebrafish infected with *M. marinum*, in which infected macrophages disseminate bacteria from the initial infection site into the body (Clay et al., 2007). In sum, *M. leprae* displays interactions with macrophages, from initial recruitment through granuloma formation, that resemble those seen for *M. marinum*. The presence of *M. leprae*-infected macrophages in the circulation of larvae mirrors findings in human leprosy (Drutz et al., 1972).

### *M. leprae* Infection Alters Myelin Structure

**Figure 2:**
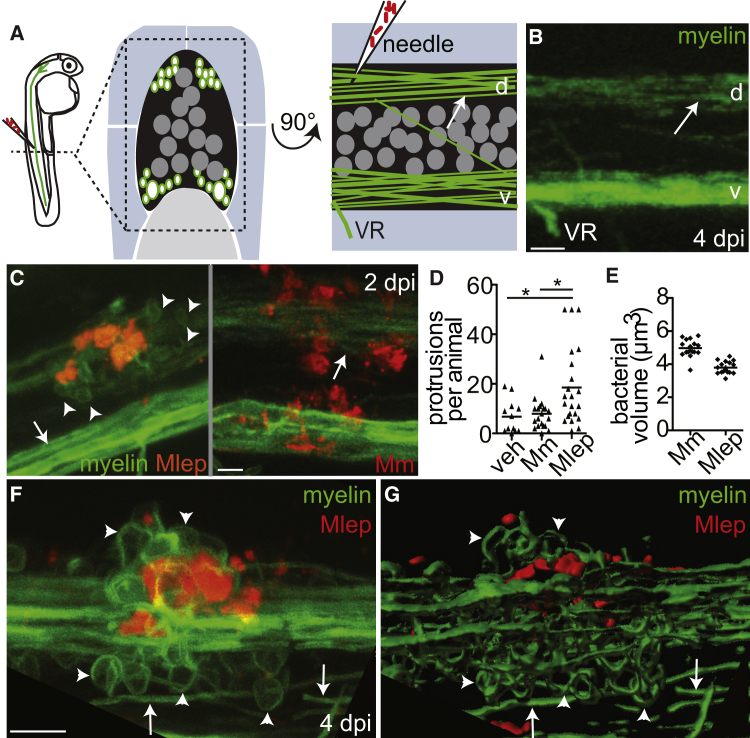
*M. leprae* Triggers Myelin Dissociation (A) Left, diagram of a spinal cord injection in an *mbp:eGFP-CAAX* larva (*mbp*), with fluorescent myelinating membrane, at 4 days post-fertilization (dpf). Transverse (middle) and sagittal (right) views of the region show the spinal cord (black), dorsal (d), and ventral (v) tracts of myelinated axons (green surrounding white axons), neuronal cell bodies (dark gray circles), and the ventral roots of spinal nerves (VR) surrounded by muscle (blue) and notochord (light gray). Arrows indicate intact myelin sheaths surrounding axons. (B) Confocal image corresponding to (A). Scale bar, 10 μm. (C) Representative confocal images of 4 dpf *mbp* larvae, 2 days post-infection (dpi) with ∼10^4^*M. leprae*, or ∼200 CFUs of *M. marinum*; arrowheads indicate myelin protrusions, quantified in (D). Scale bar, 10 μm. (D) Mean number of myelin protrusions per animal following injection with PBS vehicle (veh), *M. marinum*, or *M. leprae* (^∗^p < 0.05; one-way ANOVA, Bonferroni’s post-test). (E) Mean bacterial burden of larvae from (C); measured by fluorescent pixel intensity like in Figure 1F. (F and G) Representative confocal image (F) and rendering (G) of myelin protrusions in a 6 dpf larva 4 dpi with ∼10^4^*M. leprae* (Movie S1). Scale bar, 10 μm. See also Table S1 and Movie S1.

We next investigated the interactions of *M. leprae* with cells of the zebrafish nervous system, to determine if infection produced demyelination. Transgenic *mbp* (myelin basic protein) larvae express membrane-localized GFP that labels the myelinating membrane of glia in both the peripheral nervous system (Schwann cells) and central nervous system (oligodendrocytes) (Jung et al., 2010). Oligodendrocytes express all Schwann cell determinants that have been reported to interact with *M. leprae* (Table S1), and myelin structure is similar in the central and peripheral nervous systems (Morell and Quarles, 1999). Therefore, we studied *M. leprae* interactions with nerves in the spinal cord rather than peripheral nerves because of their easier accessibility. We injected fluorescent *M. leprae* into the dorsal spinal cord of larvae at 2–4 days post-fertilization (dpf), and imaged nerves at 4–8 dpf, a developmental stage at which these tracts have become myelinated (Czopka, 2016) (Figures 2A and 2B). At 2 dpi (4 dpf), we observed cellular protrusions from an otherwise intact myelin sheath, clustered around *M. leprae* in the nerve (Figure 2C). *M. marinum* injected into the dorsal spinal cord did not alter the myelinating membrane structure, even though the *M. marinum* burdens at the injection sites were higher than those in *M. leprae* infections (Figures 2C–2E). The *M. leprae*-induced myelin protrusions increased in size and number with time but always remained next to the bacteria (Figure 2F). Three-dimensional rendering showed that protrusions were doughnut shaped, not spherical, suggesting that these structures were not cell bodies but rather protrusions of myelinating membrane (Figure 2G; Movie S1).

### Expression of PGL-1 in *M. marinum* Confers Capacity to Alter Myelin Structure

**Figure S2:**
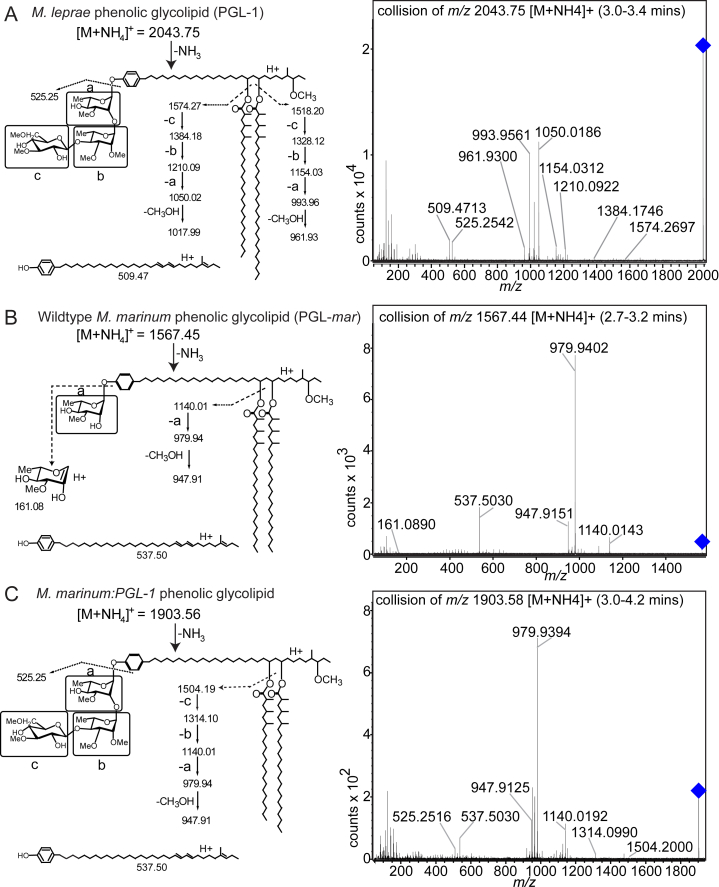
Collision-Induced Dissociation of Mycobacterial PGLs, Related to Figures 3A and 3B (A) Collision-induced dissociation of an ammoniated adduct of PGL-1 standard isolated from *M. leprae* shows calculated masses on the structure (left) with detected masses shown in the chromatogram (right). 30V collision energy; blue diamond = collided ion. Detected ions are assigned when they match the calculated masses within 10 parts per million (ppm), and the detailed substructures shown are consistent with known natural PGL components, but are not established directly. a, b and c correspond to the proximal, intermediate distal monosaccharides from *M. leprae* PGL-1. (B) Collision-induced dissociation of PGL-*mar* standard isolated from WT *M. marinum*, shown as in (A). (C) Collision-induced dissociation of PGL-1 from total lipid extract of an *M. marinum:PGL-1* log phase culture, shown as in A. The fragments at *m/z* 525.25 detected in collision of PGL-1 from *M. marinum:PGL-1* (C) and *M. leprae* (A), correspond to the known mass of PGL trisaccharides. In *M. marinum*:PGL-1 we detected PGL fragments corresponding to the loss of mycocerosic acid and monosaccharide c (*m/z* 1504.19) or the disaccharide cb (*m/z* 1314.10) or the trisaccharide cba (*m/z* 1140.01). These fragments provide a highly specific signature for the trisaccharide structure that is seen also in *M. leprae* PGL-1 fragments *m/z* 1574.27, *m/z* 1384.18 and *m/z* 1210.09 (A).

**Figure 3:**
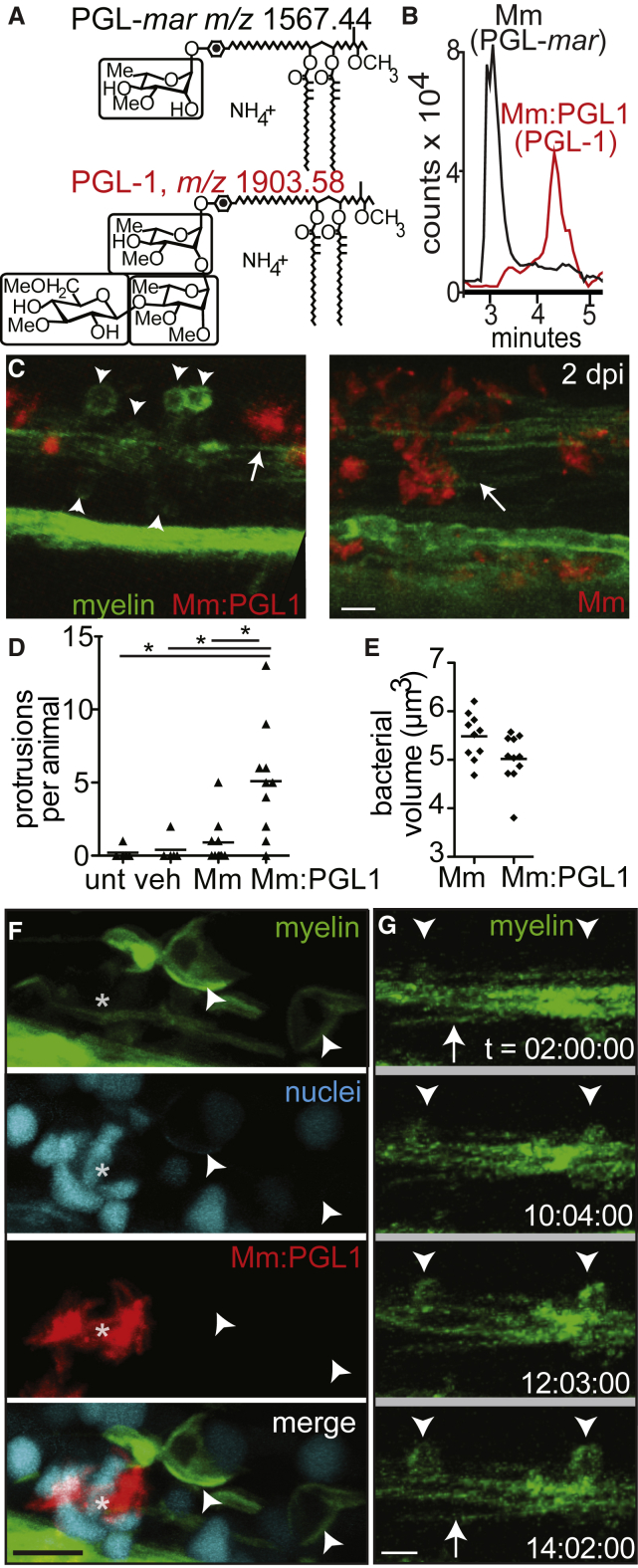
Phenolic Glycolipid 1 Triggers Myelin Dissociation (A) Normal phase high-performance liquid chromatography mass spectrometry measured at the known mass-to-charge ratios (*m/z)* for triglycosylated and monoglycosylated forms of PGL, leading to the separate detection of PGL-*mar* (*m/z* 1,567.44, upper structure) and PGL-1 (*m/z* 1,903.58, lower structure) in total lipid extracts of the indicated strains (B). (B) Chromatograms of the ions depicted in (A), showing the increased retention time of PGL-1 from *M. marinum*:PGL-1 (Mm:PGL1) compared to that of PGL-*mar* from WT *M. marinum*. (C) Representative confocal images, like in Figure 2C, of 2 dpi (4 dpf) larvae infected with ∼200 CFU *M. marinum* or *M. marinum*:PGL-1; myelin protrusions are quantified in (D). Scale bar, 10 μm. (D) Mean number of myelin protrusions per animal in uninjected larvae (unt) or after injection with PBS vehicle (veh), *M. marinum*, or *M. marinum*:PGL-1 (∼200 CFU each; ^∗^p < 0.05, one-way ANOVA with Bonferroni’s post-test). (E) Mean bacterial burden at the injection site of larvae from (D). (F) Representative confocal image of a 6 dpf larva with fluorescently labeled nuclei, 4 dpi with *M. marinum*:PGL-1 (∼100 CFU). Asterisk indicates an aggregate of infected cells. Scale bar, 10 μm. (G) Stills from time-lapse imaging of an *mbp* larva injected with *M. marinum*:PGL-1, showing myelin protrusions forming from apparently intact myelin. Arrow, intact myelin sheath; arrowheads, myelin protrusions. Relative time code. Scale bar, 10 μm. See also Figures S2 and S3 and Movie S2.

*In vitro* studies suggest that *M. leprae* interacts with glial determinants through a surface-localized long chain lipid, known as PGL-1 (*m/z* 2,043.75), which carries a unique trisaccharide (Ng et al., 2000, Renault and Ernst, 2015) (Figure S2A). The phenolic glycolipid of *M. marinum* contains a monosaccharide and shorter lipid chains that renders it detectable at a lower mass value (*m/z* 1,567.44) (Figures 3A and S2B). We wondered if the trisaccharide that is normally found on *M. leprae* PGL-1 would be sufficient to render *M. marinum* capable of altering myelin. We transformed *M. marinum* with the six *M. leprae* genes responsible for assembly of PGL-1’s terminal disaccharide (Tabouret et al., 2010). Ion chromatograms (Figures 3A and 3B) and collision-induced dissociation mass spectrometry (Figure S2) of total lipid from the transformant, *M. marinum*:*PGL-1*, proved that it produced triglycosylated PGL-1. PGL-1 expression conferred on *M. marinum* the ability to cause myelin protrusions, indistinguishable from those of *M. leprae* in both morphology and their invariable co-localization with the bacteria (Figures 3C–3E).

**Figure S3:**
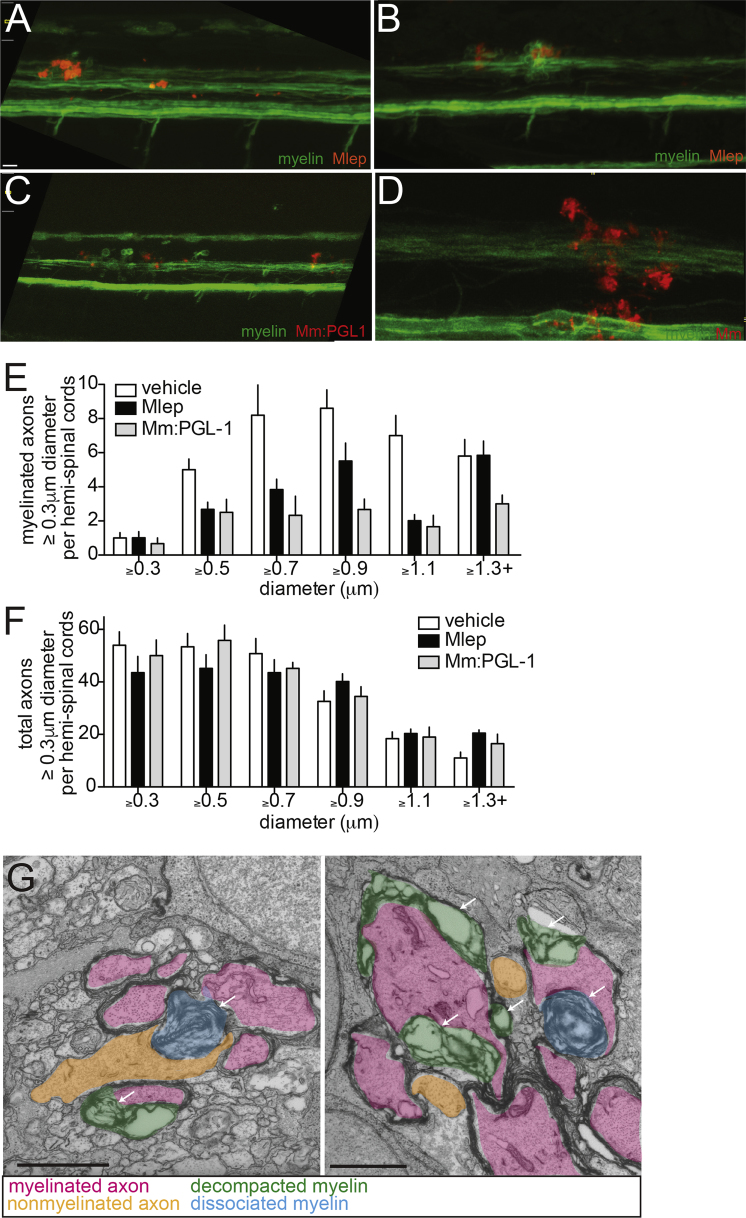
Nerve Damage in Infected Larvae, Related to Figures 3C and 4 Lower magnification views of *mbp:eGFP-CAAX* larvae at 2 dpi (4 dpf) or 4 dpi (6 dpf) with *M. leprae* and *M. marinum:PGL-1*, showing the apparently intact myelin sheath outside of the lesion (*M. marinum* shown for comparison). 10 μm bar. (A) 2 dpi, *M. leprae*. (B) 4 dpi, *M. leprae*. (C) 2 dpi, *M. marinum:PGL-1.* (D) 2 dpi, WT *M. marinum*. (E) Mean (±SEM) number of myelinated axons randomly selected (see the STAR Methods) in the hemi-spinal cords of 5 dpf larvae 2 days post-injection (dpi) with PBS control vehicle (white), *M. leprae* (black) or *M. marinum:*PGL-1 (gray). 3 larvae per group. (F) Mean (±SEM) number of total axons, quantified as in (E). (G) Two additional examples of myelin decompaction and dissociation in the spinal cords of *M. leprae*-infected fish from Figure 4B. Highlights indicate myelinated axons (pink), nonmyelinated axons (orange), decompacted myelin (green highlight and arrows), and myelin dissociated from axons (blue highlight and arrows). 10 μm bars.

The protrusions, like those produced by *M. leprae*, did not colocalize with a histone marker that labels cell nuclei. This suggested they did not simply represent an accumulation of oligodendrocyte cell bodies, but rather were composed of myelinating membrane (Figure 3F). Using time-lapse imaging to observe the formation of protrusions in real time, we observed that an intact myelin sheath near the *M. marinum*:*PGL-1* injection site began to condense and then bulge (Figure 3G). Protrusions formed by 10 hr post-infection and expanded over time (Figure 3G). To further test if myelin protrusions represent recruitment or proliferation of oligodendrocyte cell bodies, we generated larvae with a single GFP-labeled oligodendrocyte. Time-lapse movies of these larvae showed that individual oligodendrocytes form myelin protrusions by retracting portions of myelinating membrane from previously intact sheaths (Movie S2A). This occurred after injection with *M. marinum*:*PGL-1*, but not with PBS (compare Movies S2A and S2B). These findings strongly suggested that the protrusions arise from previously intact myelin sheaths, consistent with early demyelination. Similar to human leprosy (de Freitas and Said, 2013), myelin dissociation occurred in discrete areas, with the surrounding myelin sheaths remaining intact (Figures S3A–S3D).

### Transmission Electron Microscopy Shows PGL-1-Mediated Demyelination and Axonal Damage

**Figure 4:**
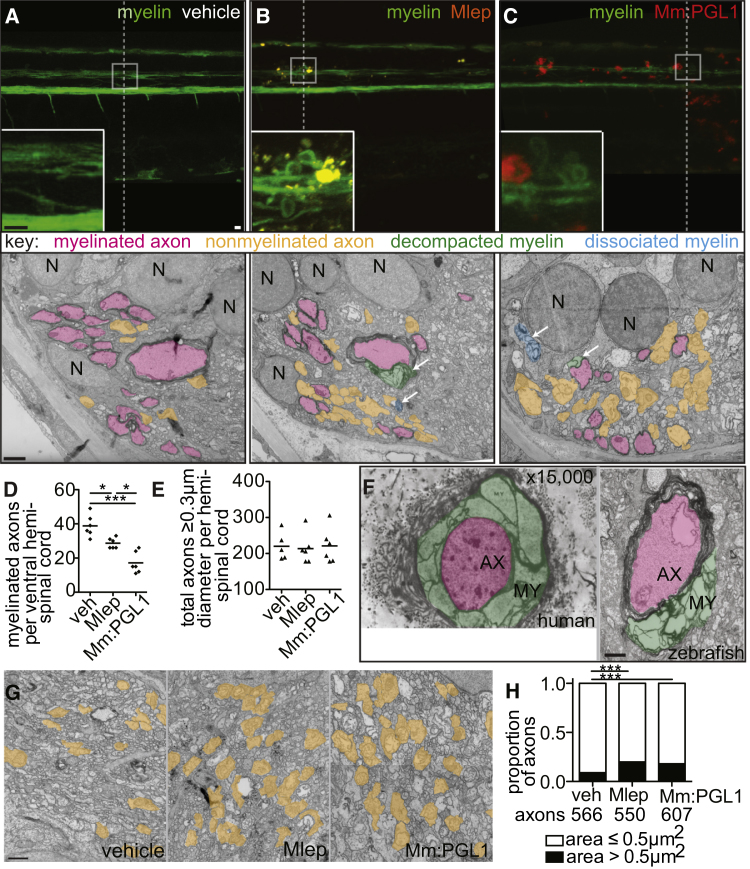
*M. leprae* Alters Nerve Ultrastructure (A–C) Representative confocal images of the spinal cord injection site (upper; scale bar, 10 μm) in *mbp* larvae at 2 dpi (5 dpf). Insets show magnifications of boxed regions; dashed lines indicate approximate location of the TEM section, shown below with 1 μm scale bars. Highlights indicate myelinated axons (pink), nonmyelinated axons (orange), decompacted myelin (green highlight and arrows), and myelin dissociated from axons (blue highlight and arrows). N, neuronal cell body. Apparent yellow in (B) is due to colocalization of red *M. leprae* and green myelin and bleed-through of the red PKH into the green channel. (D and E) Mean number of myelinated axons (D) and total axons (E) per hemi-spinal cord section in larvae injected with PBS vehicle (veh), *M. leprae*, or *M. marinum:*PGL-1. (Two hemi-spinal cords scored per larvae; three larvae per group; one-way ANOVA with Bonferonni’s post-test; ^∗^p < 0.05; ^∗∗∗^p < 0.001.) (F) Myelin decompaction in a radial nerve biopsy from a leprosy patient (left) (Job 1973, republished with permission), compared to similar alterations in the myelin of a *M. leprae*-infected larva (right). MY, myelin; AX, axon; highlights indicate myelinated axons (pink) and decompacted myelin (green); scale bar, 1 μm. (G) TEMs of larvae obtained like in (A), through matched anatomical regions. Nonmyelinated axons with diameter ≥ 0.5 μm^2^ are highlighted in orange; scale bar, 1 μm. (H) Proportion of nonmyelinated axons with area >0.5 or ≤0.5 μm^2^ from larvae obtained like in (A) (^∗∗∗^p < 0.001; Fisher’s exact test). See also Figure S3.

Demyelination can be imaged in detail by transmission electron microscopy (TEM). We compared TEM images of transverse sections through areas of myelin protrusions at 2 days after infection to identical sections through the injection site of PBS-injected fish (Figures 4A–4C). TEMs from animals injected with *M. leprae* or *M. marinum*:*PGL-1* revealed a selective decrease in myelinated axons, while the total number of axons was preserved (Figures 4D, 4E, S3E, and S3F). Higher-magnification images revealed apparently intact axons surrounded by disorganized myelin, with large spaces in between the individual lamellae (Figure S3G); this myelin decompaction is characteristic of early demyelination in human leprosy (Figure 4F) (Job, 1973, Shetty et al., 1988). The condensed, fragmented myelin, which was no longer associated with axons, was observed scattered throughout the extracellular space (Figures 4A–4C and S3G).

In vitro studies have focused on *M. leprae*-induced demyelination as a mechanism of nerve injury (Rambukkana et al., 2002, Scollard, 2008). However, the peripheral neuropathy of human leprosy involves both myelinated and nonmyelinated axons (Medeiros et al., 2016, Shetty and Antia, 1996, Shetty et al., 1988). To test if nonmyelinated axons were also affected in zebrafish, we selected an area of the spinal cord containing only one myelinated axon surrounded by many nonmyelinated axons. We observed swelling of nonmyelinated axons, as evidenced by their increased area compared to PBS-injected control (Figures 4G and 4H). Thus, *M. leprae* and *M. marinum*:*PGL-1* rapidly induce damage to both myelinated and nonmyelinated axons in the zebrafish, similar to the pathological changes found in human leprosy.

### *M. leprae-*Induced Nerve Damage Is Mediated by Macrophages

**Figure 5:**
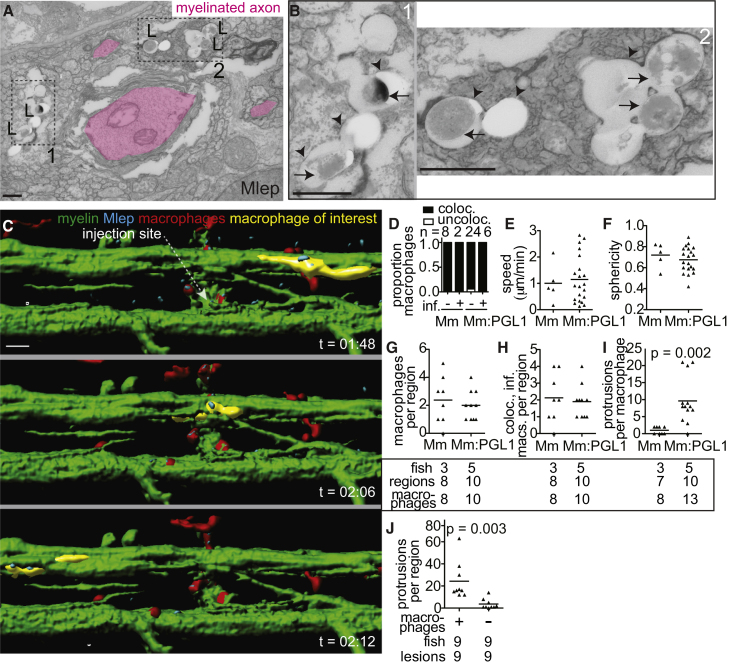
Macrophages Mediate *M. leprae* Demyelination (A) TEM from 6 dpi (8 dpf) larva showing *M. leprae* bacilli (L) within a phagocyte contacting a myelinated axon. Dashed line indicated insets 1 and 2, shown in (B); myelinated axons highlighted in pink; scale bar, 1 μm. (B) Insets from (A), showing the *M. leprae* double membrane (arrows) and phagosomal membranes (arrowheads); scale bar, 1 μm. (C) Rendered still images from a time-lapse movie (Movie S6) of an *M. leprae*-infected double-transgenic *mbp;mpeg1* larva with fluorescent macrophages and myelinating membrane. At 4 days post-fertilization, the larva was infected in the spinal cord and immediately imaged for 12 hr, revealing infected macrophages patrolling intact myelin sheaths (a myelin-patrolling, infected macrophage highlighted in yellow). Scale bar, 10 μm. (D) Proportion of uninfected (−) or infected (+) macrophages that colocalized with myelin in 4 dpf larvae infected with *M. marinum* or *M. marinum:PGL-1*. n = number of macrophages scored. (E) Mean speed of macrophages in the larvae from (D). (F) Mean sphericity of macrophages in the larvae from (D). (G) Mean number of macrophages per infected region in (5 dpf) *mbp* larvae 2 dpi with *M. marinum* or *M. marinum:PGL-1*. Numbers of fish, regions, and macrophages scored per group are indicated. (H) Mean number of macrophages per region that were both infected and myelin colocalized in the larvae from (G). (I) Mean number of myelin protrusions per macrophage in the larvae from G. Student’s t test. (J) Myelin protrusions per *M. leprae*-infected region in WT *mbp* larvae (+) or those depleted of macrophages (−) by injection with *irf8* morpholino and lipo-clodronate. Student’s t test. Data are representative of at least two separate experiments. See also Figure S4 and Movies S3, S4, S5, S6, S7, and S8.

Contrary to the previous model (Rambukkana, 2000), our findings in vivo did not support contact or infection of glia by *M. leprae* early in infection. We did not observe mycobacteria within myelin protrusions by confocal microscopy (Figure 2F), nor did we observe bacteria in direct contact with myelin or infected glia by TEM. All observed bacteria were within phagosomes of macrophages abutting the axons (Figures 5A and 5B). Given the presence of macrophages in the demyelinating lesions, we wondered if infected macrophages, rather than bacteria directly, initiated demyelination and nerve damage. Three findings in human leprosy support this idea: (1) macrophages, including those harboring *M. leprae*, are abundant in affected nerves even early in disease (Job, 1973, Pandya and Antia, 1974, Shetty and Antia, 1996, Shetty et al., 1988). (2) Early stages of demyelination feature vacuolar myelin structures, in which the lamellae have split and separated (Job, 1973), associated with infected macrophages beneath the basement membrane of Schwann cells. (3) The unique trisaccharide of *M. leprae* PGL-1 confers both demyelinating (Ng et al., 2000, Renault and Ernst, 2015) and macrophage-modulating effects in vitro (Manca et al., 2012, Tabouret et al., 2010). The plausibility of a macrophage-induced mechanism is further supported by findings that macrophages mediate demyelination and nerve damage in multiple sclerosis and Guillain-Barré syndrome (Bogie et al., 2014, Martini et al., 2008, Martini and Willison, 2016, Nikić et al., 2011).

Macrophages are associated with nerves under homeostatic conditions in humans and rodents, both in the peripheral and central nervous systems (Kierdorf et al., 2013, Klein and Martini, 2016, Müller et al., 2010). In the case of nerve injury, their numbers increase (Klein and Martini, 2016), presumably because they play roles in scavenging debris and repair. In the zebrafish too, we observed macrophages arriving from the blood and patrolling axons in uninjected larvae, and their numbers increased in response to the trauma of PBS injection (Movies S3, S4, and S5).

**Figure S4:**
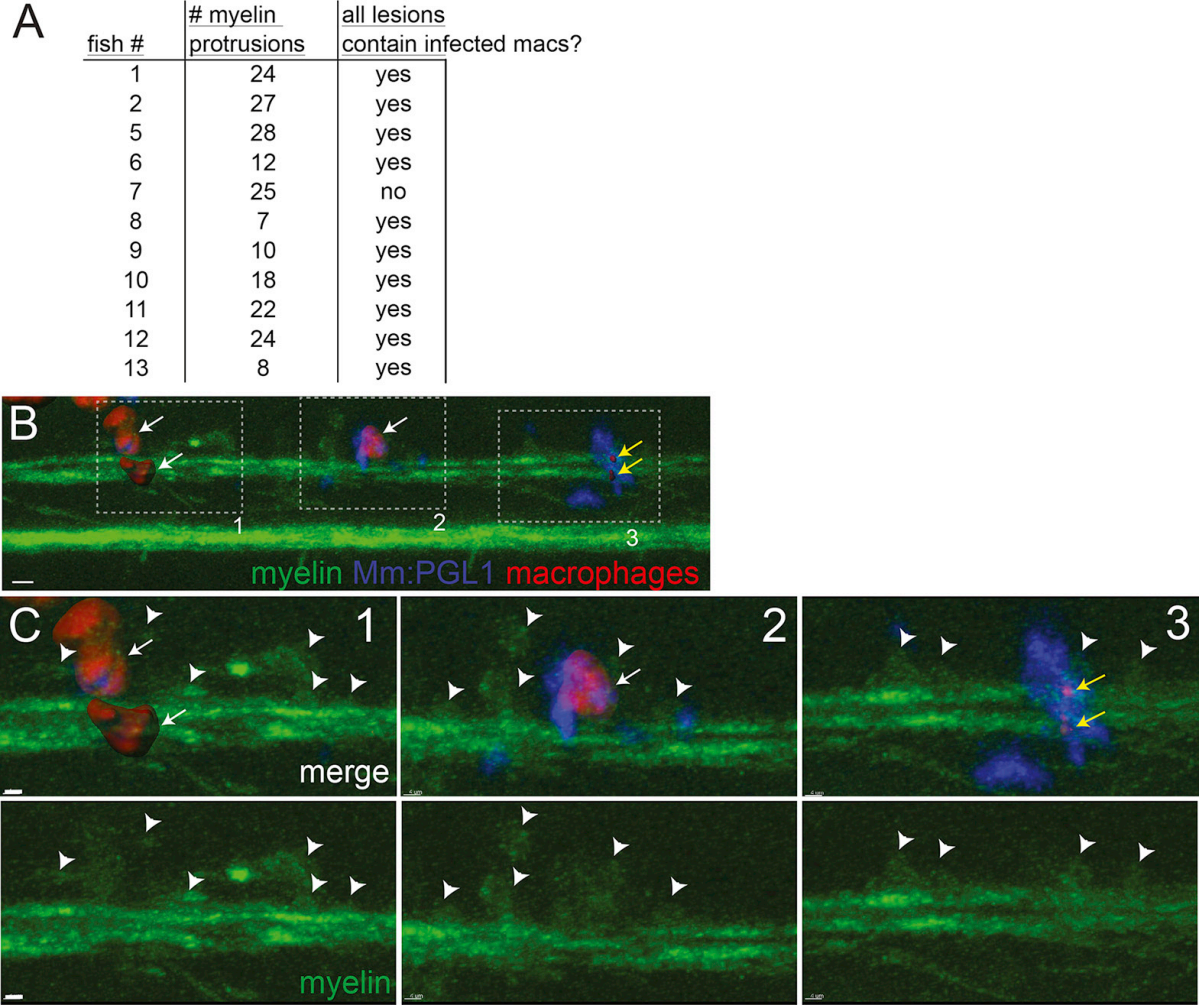
Association of Infected Macrophages with Demyelinating Lesions, Related to Figure 5I At 2 days post-infection (5 dpf), nerve lesions were identified in *M. marinum*:*PGL-1*-infected *mbp:eGFP-CAAX; mpeg1:Brainbow* larvae (Figure 5I). (A) Each lesion was scored for presence or absence of infected macrophages; association of infected macrophages with myelin protrusions was deemed significant (p = 0.01) with a two-tailed binomial test. (B) Lesion from fish #7 (A), which had 3 demyelinating lesions (dashed boxes). For clarity, red macrophages or fragments of their red membrane are shown as rendered objects; green myelin and blue bacteria are show as the unmodified fluorescent images. 10 μm bar. (C) Insets of each lesion from B, showing myelin protrusions and colocalized, infected macrophages that are still intact (1 and 2) or fragmented (3) and dead. Arrowheads indicate myelin protrusions; for clarity, not every myelin protrusion that was scored is indicated with an arrowhead. White arrows, intact macrophages; yellow arrows, fragments of dsRed-positive macrophage membrane. 10 μm bars.

We asked if infection with PGL-1-expressing bacteria made these macrophages capable of demyelinating axons. We used blue or far-red fluorescent bacteria to infect transgenic larvae with green fluorescent myelinating membrane and red fluorescent macrophages. Immediately after infection, macrophages were recruited to the injection site, entered the spinal cord, and phagocytosed the majority of the bacteria; this was equally the case for *M. leprae*, *M. marinum* and *M. marinum*:*PGL-1* (Movies S6, S7, and S8). Moreover, in the context of each infection, macrophages, whether or not infected, patrolled the axons, assuming a flattened, elongated shape as they moved between them (Figure 5C; Movies S6, S7, and S8). We noted that some infected macrophages moved more slowly and eventually became sessile within the first 12 hours, resulting in prolonged intimate contact with the myelin in discrete areas. This slowing down of infected macrophages has been noted in *M. marinum* granulomas (Davis and Ramakrishnan, 2009). Here, too, we observed more slowly moving infected macrophages in the context of all three infections, suggesting it was an infection-dependent, but not PGL-1-dependent, phenomenon (Movies S6, S7, and S8). This was confirmed by a quantitative comparison of macrophage behavior during the first 12 hours following *M. marinum* versus *M. marinum*:*PGL-1* infection*:* there were no differences in macrophage speed, shape, or tendency to associate with myelin (Figures 5D–5F). Macrophage co-localization with myelin continued to be similar between the two bacterial groups at 2 days post-infection, when demyelination begins (Figures 5G and 5H). Yet, only colocalization of *M. marinum*:*PGL-1*-infected macrophages with myelin produced myelin protrusions (Figure 5I). All demyelinating lesions were associated with macrophages in 10 of 11 animals scored (Figure S4; p = 0.01, two-tailed binomial test with an expected 0.5 frequency). In the 11th animal, 2 of the 3 demyelinating lesions were associated with macrophages, while the third had defined clusters of bacteria with residual fluorescent macrophage membrane, suggesting that the co-localized, infected macrophage had died (Figures S4B and S4C). Finally, to directly test if macrophages were required for *M. leprae*-induced demyelination, we created macrophage-depleted fish by administering an *irf8* morpholino followed by clodronate liposomes (Pagán et al., 2015). Macrophage depletion reduced myelin protrusions by 85% in *M. leprae*-infected larvae, confirming the essential role of macrophages in early demyelination (Figure 5J).

### A Theoretical Framework for the Mechanism of PGL-1- and Macrophage-Dependent Demyelination

The demyelination in leprosy is analogous to that of Gullain-Barré syndrome and multiple sclerosis, in which macrophage production of reactive oxygen species (ROS) and reactive nitrogen species (RNS) can trigger swelling and destruction of mitochondria and axons, contributing to demyelination (Bogie et al., 2014, Kiefer et al., 2001). As with leprosy, multiple sclerosis affects both myelinated and nonmyelinated axons. Similarly, the macrophages present in leprosy nerve biopsies express inducible nitric oxide synthase (iNOS) and contain nitrotyrosine, a stable end product of nitric oxide production (Lockwood et al., 2011, Schön et al., 2004). Moreover, recent work shows that mitochondria are swollen and damaged in both myelinated and nonmyelinated axons (Medeiros et al., 2016). Together, these findings suggest a model in which PGL-1 induces iNOS expression in infected macrophages, resulting in damage to mitochondria of adjacent axons. This model generates three testable predictions: (1) PGL-1-expressing bacteria induce production of iNOS and nitric oxide in the macrophages they infect; (2) PGL-1-induced nerve damage is nitric oxide dependent; and (3) nerve damage is linked to mitochondrial damage, which is also PGL-1 dependent.

### Nitric Oxide Production by Macrophages in Response to PGL-1 Mediates Demyelination

**Figure S5:**
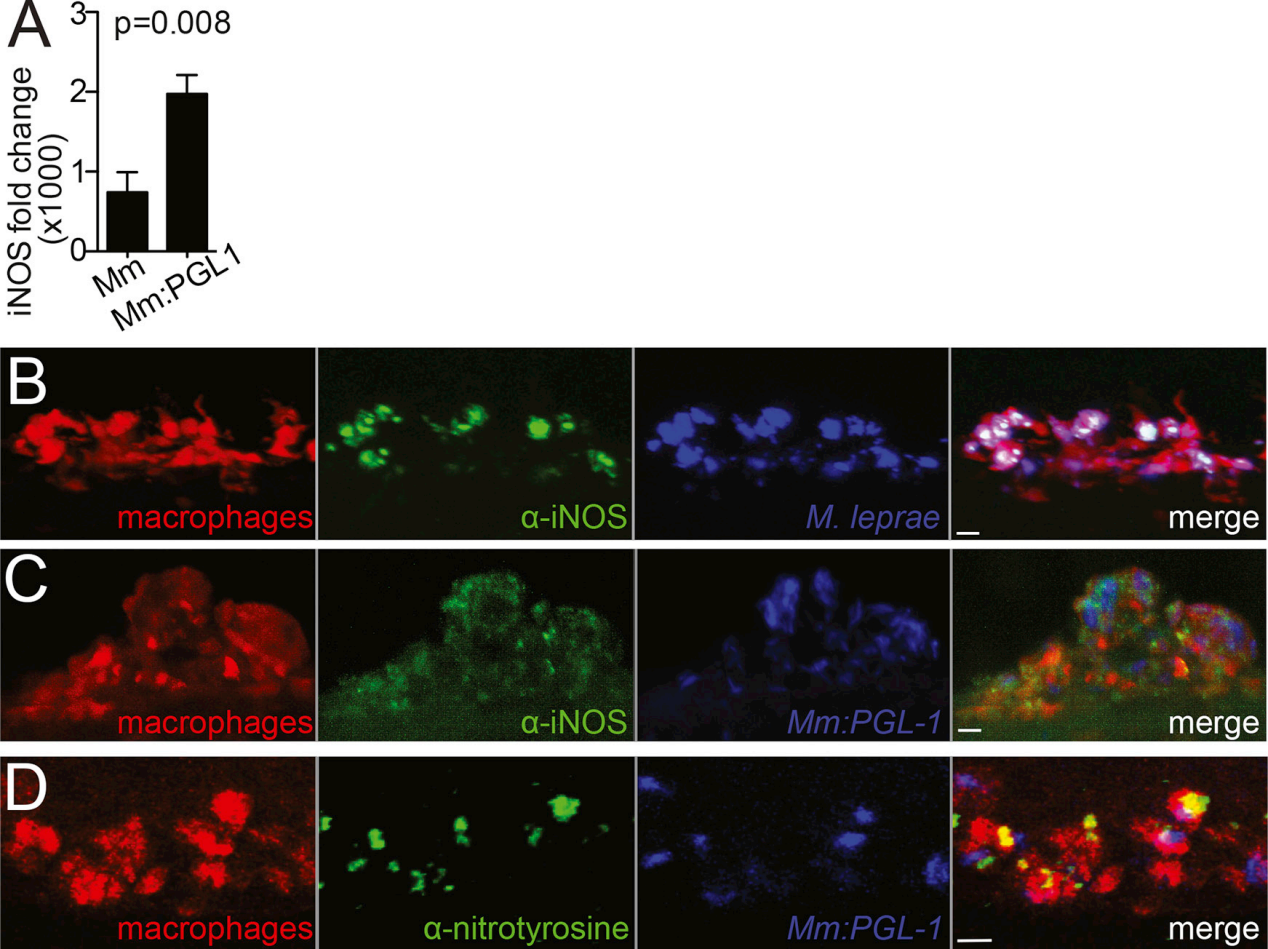
Nitric Oxide Production in Infected Macrophages, Related to Figure 6 (A) Mean (±SEM) fold change of *Nos2* (iNOS) transcript in WT murine macrophages, 6 hr after infection with WT *M. marinum* or *M. marinum:PGL-1* (both MOI 1), compared to uninfected cells. (Average of 3 independent experiments; Student’s t test). (B) Representative confocal images of a macrophage aggregate in an *mpeg1* larva infected with *M. leprae* and stained with α-iNOS antibody. 10 μm bar. (C) Representative confocal images of a macrophage aggregate in an *mpeg1* larva infected with *M. marinum:PGL-1* and stained with α-iNOS antibody. 10 μm bar. (D) Representative confocal images of a macrophage aggregate in larvae as in C, stained with α-nitrotyrosine antibody. 10 μm bar.

**Figure 6:**
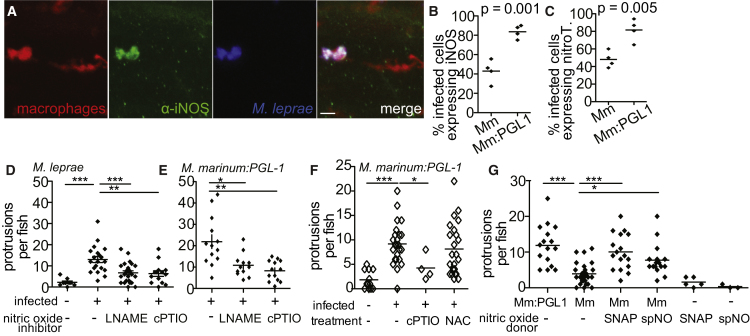
Nitric Oxide Is Necessary for Early Demyelination (A) Representative confocal images of a macrophage aggregate in the spinal cord of an *mpeg1* larva infected with *M. leprae* and stained with α-iNOS antibody. Scale bar, 10 μm. (B) Mean percentage of infected *mpeg1*-positive macrophages that also express iNOS in 7 dpf larvae 5 dpi with WT *M. marinum* or *M. marinum:PGL-1*. (Student’s t test.) (C) Mean percentage of infected *mpeg1*-positive macrophages that stain with α-nitrotyrosine antibody (nitroT) in larvae like in (B). (Student’s t test). (D) Mean number of myelin protrusions per animal in 5 dpf *mbp* larvae 2 dpi with *M. leprae*, which were treated with 0.5% DMSO vehicle (-), iNOS inhibitor (L-NAME), or ROS/RNS scavenger (cPTIO). (^∗∗^p < 0.01; ^∗∗∗^p < 0.001; one-way ANOVA with Dunnett’s multiple comparison test.) (E) Mean number of myelin protrusions per animal in 5 dpf *mbp* larvae 2 dpi with *M. marinum:PGL-1*, treated like in (D). (^∗^p < 0.05; ^∗∗^p < 0.01; one-way ANOVA with Dunnett’s multiple comparison test.) (F) Mean number of myelin protrusions per animal in 5 dpf *mbp* larvae 2 dpi with *M. marinum:PGL-1*, which were treated with 0.5% DMSO vehicle (“-”), nitric oxide scavenger (cPTIO), or ROS scavenger NAC. (^∗^p<0.05; ^∗∗∗^p<0.001; one-way ANOVA with Dunnett’s multiple comparison test.) (G) Mean number of myelin protrusions per animal in larvae infected like in (F), which were soaked post-injection in 0.5% DMSO vehicle (“-”) or in nitric oxide donors SNAP or spermine NONOate (spNO). (^∗^p<0.05; ^∗∗∗^p<0.001; one-way ANOVA with Dunnett’s multiple comparison test.) See also Figure S5.

To test the first prediction of our model, we asked if PGL-1 induces *Nos2* (the gene that encodes iNOS) in cultured murine bone marrow-derived macrophages. *M. marinum:PGL-1* induced 2.8-fold more *Nos2* in macrophages than wild-type *M. marinum*, showing a substantial contribution from PGL-1 (Figure S5A). In the zebrafish too, *M. leprae*- or *M. marinum*:PGL-1-infected macrophages were iNOS and/or nitrotyrosine positive, both in the periphery and in the nervous system (Figures 6A and S5B–S5D). Again, *M. marinum:*PGL-1 infection was associated with more iNOS- and nitrotyrosine-positive macrophages than wild-type *M. marinum* (Figures 6B and 6C). Thus, PGL-1-expressing mycobacteria induce macrophages to produce nitric oxide through transcriptional induction of iNOS.

To test the second prediction of our model, we asked if nitric oxide induces early demyelination by treating infected fish with the iNOS inhibitor L-NAME or the nitric oxide scavenger cPTIO. Both treatments inhibited demyelination, in larvae infected with *M. leprae* or *M. marinum:*PGL-1 (Figures 6D and 6E). Two RNS, nitric oxide and peroxynitrite, have been implicated in damage to axons and myelin (Smith et al., 1999). Formation of peroxynitrite requires superoxide anion, a ROS. To differentiate between damage caused by nitric oxide and by peroxynitrite, we treated *M. marinum*:PGL-1*-*infected larvae with NAC, a scavenger of ROS. Demyelination was not significantly reduced in NAC-treated animals, implicating nitric oxide, rather than peroxynitrite, as the primary contributor to demyelination. Further, the nitric oxide donors SNAP and spermine NONOate induced demyelination in larvae infected with wild-type *M. marinum* (Figure 6G). Notably, nitric oxide donors failed to cause demyelination in the absence of *M. marinum* infection (Figure 6G). The most likely explanation for this is that the amount of nitric oxide released by the donors is insufficient to produce demyelination. *M. marinum* induces iNOS and nitric oxide in macrophages, but this is insufficient for demyelination. The nitric oxide produced by the donors and *M. marinum* together may cross the threshold required to produce demyelination. Alternatively, nitric oxide may act in concert with one or more additional macrophage determinants that are induced by any virulent mycobacterium.

### PGL-1-Induced Axonal Damage Is Associated with Mitochondrial Swelling and Loss

**Figure 7:**
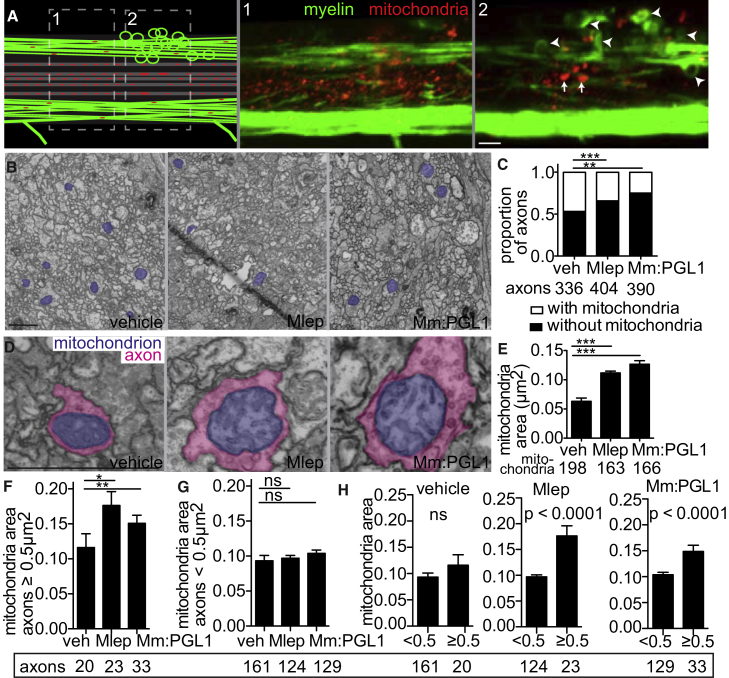
*M. leprae* Infection Damages Axonal Mitochondria (A) Diagram (left) of a demyelinating lesion in a 2 dpi (5 dpf) *mbp* larva with fluorescent mitochondria in axons. Dashed boxes indicate insets 1 and 2, with corresponding confocal images, showing mitochondria outside the lesion (inset 1) and those within the lesion (inset 2). Arrowheads indicate myelin protrusions; arrows indicate enlarged mitochondria. Scale bar, 10 μm. (B) Representative TEMs of matched anatomical regions showing the number of axon mitochondria (purple) in larvae injected with PBS, *M. leprae*, or *M. marinum:PGL-1*. Scale bar, 1 μm. (C) Proportion of axons with mitochondria to those without, in larvae like in (B); the number of axons scored are listed below (contingency analysis corrected for multiple comparisons; ^∗∗^p = 0.004; ^∗∗∗^p < 0.0002). (D) Representative TEMs of enlarged mitochondria (purple) within enlarged axons (pink), in larvae like in (B). Scale bar, 1 μm. (E) Mean (±SEM) area of mitochondria in axons, in larvae like in (B); number of mitochondria scored are listed below. (^∗∗∗^p < 0.001; one-way ANOVA with Dunnett’s multiple comparison.) (F) Mean (±SEM) area of mitochondria in nonmyelinated axons with area ≥0.5 μm^2^, in larvae like in (B) (^∗^p < 0.05, ^∗∗^p < 0.01; one-way ANOVA with Dunnett’s multiple comparison). (G) Mean (±SEM) area of mitochondria in nonmyelinated axons with area <0.5 μm^2^, in larvae like in (B) (one-way ANOVA with Dunnett’s multiple comparison). (H) Data from (F) and (G) are displayed per experimental group, showing mean (±SEM) area of mitochondria in large versus small nonmyelinated axons (one-way ANOVA with Dunnett’s multiple comparison).

The third prediction of our model is that mitochondrial damage is linked to nerve damage, and is dependent on PGL-1 production by bacteria. Confocal microscopy of *mbp* larvae expressing a fluorescent protein in axonal mitochondria (neuronal tubulin promoter driving expression of dsRed protein with a mitochondrial signal sequence; see the STAR Methods) revealed both mitochondrial swelling and selective loss in regions close to demyelinating lesions (Figure 7A). TEMs through demyelinating lesions of *M. leprae* and *M. marinum*:PGL-1-infected larvae had fewer axonal mitochondria compared to PBS-injected larvae (Figures 7B and 7C). The remaining mitochondria were enlarged in infected larvae compared to PBS-injected controls, similar to the mitochondrial swelling reported for leprosy and multiple sclerosis (Medeiros et al., 2016, Nikić et al., 2011) (Figures 7D and 7E). If mitochondrial damage is linked to axonal damage, then it should be most prevalent in swollen axons. Two analyses showed that this was the case: first, the increase in mitochondrial area in infection over PBS control occurred in axons with an area ≥0.5 μm^2^, but not in those with an area <0.5 μm^2^ (Figures 7F and 7G). Second, within each of the three cohorts, mitochondrial area was increased only in the large axons (≥0.5 μm^2^) of *M. leprae* and *M. marinum*:PGL-1-infected larvae, not in PBS-injected larvae (Figure 7H). As expected, there was no difference in mitochondrial area in the axons of PBS-treated animals, where the differences in axon size reflect normal physiological variation, rather than pathology. Collectively, these findings support the model that reactive nitrogen species produced by infected macrophages damage axonal mitochondria and cause demyelination.

## Discussion

Our work suggests a mechanism for the earliest nerve injury associated with leprosy: over-exuberant production of nitric oxide by macrophages, in response to the *M. leprae*-specific PGL-1, damages axonal mitochondria and initiates demyelination. Phenolic glycolipids likely evolved to increase infectivity by recruiting macrophage subsets that are particularly permissive to mycobacterial infection (Cambier et al., 2014). In an accompanying study, we show that PGL induces tissue-resident macrophages that first phagocytose infecting bacteria to express CCL2, which recruits permissive macrophages to the site of infection enabling mycobacteria to transfer from the microbicidal first-responding tissue macrophages into the recruited growth-permissive monocytes (Cambier et al., 2017). In this paper, we find that the specialized, triglycosylated form of *M. leprae* PGL-1 retains this basal role, while acquiring additional macrophage-modulating functions that produce demyelination. PGL-1 has been found to alter inflammatory mediator expression in cultured macrophages (Manca et al., 2012, Tabouret et al., 2010), and our work now assigns a central role for this immunomodulation in early leprosy neuropathy.

In terms of the relevance of our findings to human leprosy, macrophages, often infected, are a consistent presence within early nerve lesions of leprosy patients (Job, 1973, Pandya and Antia, 1974, Shetty and Antia, 1996, Shetty et al., 1988). Furthermore, iNOS upregulation has been reported in both “pro-inflammatory” paucibacillary and “anti-inflammatory” multibacillary leprosy lesions (Lockwood et al., 2011, Teles et al., 2013), both of which are associated with nerve damage. As to how infected macrophages might reach nerves, one way is by direct seeding from a skin granuloma into an underlying nerve trunk. In support of this possibility, a leprosy cohort study found that the most significant risk factor for development of neuropathy in a peripheral nerve was the presence of an overlying skin lesion (van Brakel et al., 2005). A second possibility is through hematogenous dissemination. This work and others suggest that circulating macrophages patrol axons under homeostatic conditions, reaching axons by extravasating from local blood vessels (Klein and Martini, 2016). Bacteremia is common in leprosy patients, with circulating bacteria found in mononuclear phagocytes (Drutz et al., 1972, Lane et al., 2006) and in blood vessels of apparently normal skin (Ganapati and Chulawala, 1976). We suggest that infected macrophages have a similar propensity to reach nerves and patrol them to their uninfected counterparts. Some may slow down and stall as *Mycobacterium*-infected macrophages are wont to do (Davis and Ramakrishnan, 2009). The resultant prolonged intimate contact with the nerve may initiate damage through the mechanism we have uncovered. This hematogenous dissemination model predicts that *M. leprae*-infected macrophages are widely distributed in nerves. Indeed, biopsies of the apparently normal skin of leprosy patients find subclinical, diffuse neuropathy in conjunction with infected macrophages (Ganapati et al., 1972, Pandya and Antia, 1974). Finally, household contacts of leprosy patients are significantly more likely to have *M. leprae* DNA in their peripheral blood than non-contacts, and longitudinal follow-up shows that these individuals are more likely to develop leprosy (Wen et al., 2013), suggesting that hematogenous dissemination of *M. leprae* is a very early and significant step in the pathogenesis of peripheral nerve damage.

Our finding that both myelinated and nonmyelinated axons are damaged by this mechanism further suggests its relevance to human leprosy neuropathy, which affects both types of axons (Medeiros et al., 2016, Shetty et al., 1988). Moreover, nonmyelinated cutaneous nerve endings are often affected early in infection, even before neurological symptoms appear (de Freitas and Said, 2013, Ganapati et al., 1972, Pandya and Antia, 1974). The idea that demyelination is a pathological manifestation, rather than a cause of nerve injury, has gained traction in the context of other demyelinating diseases, such as multiple sclerosis (Nikić et al., 2011).

The older model, which explains the neurotropism of *M. leprae* by evoking direct binding of PGL-1 to Schwann cell laminin α2 (Ng et al., 2000), is problematic in at least four ways. First, mycobacterial species that lack PGL-1 and fail to cause neuropathy are, nevertheless, able to bind laminin α2 (Marques et al., 2001). Second, the model does not explain how *M. leprae*, a nonmotile bacterium, reaches Schwann cells. Third, by requiring high bacterial burdens within Schwann cells to cause demyelination, the model fails to explain the clinical findings of nerve damage very early in infection, when only a few bacteria are present in nerve lesions. Fourth, the earliest nerve impairment in leprosy is in thermal sensation, which is mediated by nonmyelinated fibers (de Freitas and Said, 2013, van Brakel et al., 2003). Our findings resolve these inconsistencies by showing that it is the PGL-1-stimulated macrophages that initiate damage to nerves regardless of their myelination. This early innate immune-mediated nerve injury may then progress by distinct mechanisms in multibacillary and paucibacillary leprosy. In the face of an inadequate adaptive immune response in multibacillary leprosy, the inability of macrophages to control bacterial growth may result in their death, releasing bacteria into the extracellular milieu of the nerve. These released bacteria could then be taken up by Schwann cells. In paucibacillary leprosy, the onset of an adaptive immune response may enable infected macrophages to control intracellular *M. leprae*, while further enabling, or even enhancing, their neuropathological response (Scollard, 2008). This may be through the induction of pro-inflammatory cytokines such as interferon-γ (Teles et al., 2013), which may act by further stimulating reactive nitrogen species, or by engaging distinct mechanisms.

Production of nitric oxide by macrophages and other myeloid cells has been implicated in mitochondrial dysfunction and subsequent axonal injury in multiple sclerosis and Guillain-Barré syndrome (Bogie et al., 2014, Kiefer et al., 2001). Our work may offer insights into these and other neurodegenerative diseases in which myeloid cells are increasingly recognized as contributing to neuropathology (Thompson and Tsirka, 2017), as well as provide an experimental system in which to explore them.

## STAR★Methods

### Key Resources Table

**Table:** 

REAGENT or RESOURCE	SOURCE	IDENTIFIER
**Antibodies**		

Anti-iNOS (clone 54)	BD Biosciences	Cat#610431
Anti-nitrotyrosine	Merck Millipore	Cat#06-284

**Bacterial and Virus Strains**		

*M. marinum* M strain transformed with pMSP12:tdTomato, pMSP12:wasabi or pMSP12:eBFP	Cosma et al., 2006	derivatives of ATCC #BAA-535
*M. marinum:PGL-1* transformed with pMSP12:tdTomato, pMSP12:wasabi or pMSP12:eBFP	this paper	N/A
Fluorescent-stained *M. leprae,* strain Thai53	Lahiri et al., 2005	N/A
*P. aeruginosa* PAO1 expressing GFP	Brannon et al., 2009	N/A

**Chemicals, Peptides, and Recombinant Proteins**		

PBS liposomes	clodronateliposomes.org	N/A
Clodronate liposomes	clodronateliposomes.org	N/A
cPTIO (carboxy-α-phenyltetramethylnitronyl nitroxide)	Sigma	CAS # 148819-94-7
L-NAME (N_ω_-nitro-L-arginine methyl ester)	Sigma	CAS # 51298-62-5
NAC (n-acetyl-L-cysteine)	Sigma	CAS # 616-91-1
Spermine NONOate	Cayman Chemical	CAS # 136587-13-8
SNAP (*S*-nitroso-*N*-acetylpenicillamine)	Thermo Fisher	Cat # N7892
PGL-1 standard isolated from *M. leprae*	BEI Resources	Cat # NR 19342
PGL-*mar* standard isolated from wildtype *M. marinum*	this paper	N/A

**Experimental Models: Cell Lines**		

Bone-marrow derived macrophages from C57BL/6 mice	Jackson Laboratory	Stock# 000664

**Experimental Models: Organisms/Strains**		

Zebrafish: wildtype AB	University of Washington	ZFIN ID: ZDB-GENO-960809-7
Zebrafish: Tg(*mpeg1:Brainbow*)^w201^	Pagán et al., 2015	ZFIN ID: ZDB-FISH-151204-7
Zebrafish: Tg(*mbp:CAAX-GFP*)^ue2Tg^	Almeida et al., 2011	ZFIN ID: ZDB-FISH-150901-26749
Zebrafish: Tg(*kdrl:dsRed*)^s843^	Jin et al., 2005	ZFIN ID: ZDB-FISH-150901-14755
Zebrafish: Tg(*lysC:EGFP*)^nz117^	Hall et al., 2007	ZFIN ID: ZDB-FISH-150901-28454
Zebrafish: Tg(*mpeg1:YFP*)^w200Tg^	Roca and Ramakrishnan, 2013	ZFIN ID: ZDB-FISH-150901-6828

**Oligonucleotides**		

nos2 (iNOS) mRNA forward primer, sequence: CAGCTGGGCTGTACAAACCTT	Ramirez-Carrozzi et al., 2009	N/A
nos2 (iNOS) mRNA reverse primer, sequence: CATTGGAAGTGAAGCGTTTCG	Ramirez-Carrozzi et al., 2009	N/A
beta actin mRNA forward primer, sequence: AGAGGGAAATCGTGCGTGAC	Ramirez-Carrozzi et al., 2009	N/A
beta actin mRNA reverse primer, sequence: CAATAGTGATGACCTGGCCGT	Ramirez-Carrozzi et al., 2009	N/A
*irf8* morpholino, sequence: AATGTTTCGCTTACTTTGAAAATGG	Li et al., 2011	N/A
*pu.1* morpholino component 1, sequence: CCTCCATTCTGTACGGATGCAGCAT	Clay et al., 2007	N/A
*pu.1* morpholino component 2, sequence: GGTCTTTCTCCTTACCATGCTCTCC	Clay et al., 2007	N/A
*ccr2* morpholino, sequence: AACTACTGTTTTGTGTCGCCGAC	Cambier et al., 2014	N/A
*myD88* morpholino, sequence: GTTAAACACTGACCCTGTGGATCAT	Bates et al., 2007	N/A

**Recombinant DNA**		

histone labeling plasmid H2B-CFP	Megason, 2009	Addgene # 53748
Tol2 plasmid nbt-GAL4	this paper	N/A
Tol2 plasmid UAS-MLS-dsRed	O’Donnell et al., 2013	N/A
Tol2 plasmid mbp:eGFP-CAAX	Almeida et al., 2011	N/A
pWM122 plasmid with *M. leprae* PGL-1 genes	Tabouret et al., 2010	N/A

**Software and Algorithms**		

Imaris	Bitplane	N/A
ImageJ	Abramoff et al., 2004	N/A
FPC (ImageJ); macro for quantification of bacterial burden by fluorescence imaging	Takaki et al., 2013	N/A

### Contact for Reagent and Resource Sharing

Further information and requests for resources and reagents should be directed to and will be fulfilled by the Lead Contact, Lalita Ramakrishnan (lr404@cam.ac.uk).

### Experimental Model and Subject Details

Zebrafish husbandry and experiments were conducted in compliance with guidelines from the U.S. National Institutes of Health and approved by the University of Washington Institutional Animal Care and Use Committee, the Office of Animal Research Oversight of the University of California Los Angeles, and the Institutional Biosafety Committee of the University of California Los Angeles. WT AB strain zebrafish or transgenics in the AB background were used, including Tg(*kdrl:dsRed*)^s843^ (Jin et al., 2005), Tg(*mbp:CAAX-GFP*)^ue2Tg^ (Almeida et al., 2011), Tg(*mpeg1:Brainbow*)^w201^ (Pagán et al., 2015), Tg(*lysC:EGFP*)^nz117^ (Hall et al., 2007) and Tg(*mpeg1:YFP*)^w200^ (Roca and Ramakrishnan, 2013). Larvae were anesthetized with 0.02% buffered tricaine, (MS-222, Sigma) as described (Takaki et al., 2013), prior to imaging or infection. Larvae of indeterminate sex were infected by injection into the caudal vein or hindbrain ventricle at 2 dpf using a capillary needle containing bacteria diluted in PBS + 2% phenol red (Sigma), as previously described (Takaki et al., 2013), or infected in the ventral spinal cord adjacent to the cloaca at 2-4 dpf. Titered, single-cell suspensions were prepared for all *M. marinum* strains prior to infection by passing cell pellets from mid-log phase cultures (OD_600_ = 0.5 ± 0.1) repeatedly through a syringe to remove clumps, as described (Takaki et al., 2013). When two different bacterial strains were compared, several groups of larvae (n = 20 or more) were infected with different dilutions of each strain; on the day of the comparison, equivalently-infected groups of larvae (as determined by FPC) were used to assure the comparison was not biased by *in vivo* growth differences between the two strains. After infection, larvae were housed at 28.5°C, in fish water containing 0.003% PTU (1-phenyl-2-thiourea, Sigma) to prevented pigmentation.

### Method Details

Drugs were administered by adding them to the fish water; fresh drug (or DMSO vehicle for control fish) was added every 12 hr. To assess drug treatment in infected fish, equivalently-infected sibling larvae were mixed in a petri dish and held at 28.5°C for 4-6 hr after injection to allow macrophage recruitment to the injection site; larvae were then randomly allocated to the drug-treated or control group (0.5% DMSO). All drugs were dissolved in DMSO (dimethyl sulfoxide, Sigma), such that the final concentration in fish water was 0.5% DMSO. L-NAME (1000 μM), cPTIO (500 μM) or NAC (40 μM) were used to inhibit iNOS and scavenge reactive oxygen/nitrogen species, as described (Cambier et al., 2014, Roca and Ramakrishnan, 2013). SNAP (100 μM) and spermine NONOate (10 μM) were used to exogenously add nitric oxide, as described (Kong et al., 2016, Siamwala et al., 2012).

To detect iNOS or nitrotyrosine in infected larvae, equivalently-infected larvae were euthanized by tricaine overdose, fixed overnight at 4°C in 4% paraformaldehyde (Sigma) + 4% sucrose (Fisher), permeabilized for 30 min in PBST (PBS + 0.5% Triton X-100 (Sigma)), then stained overnight at 4°C in iNOS or nitrotyrosine antibodies (see Key Resources Table) diluted 1:200, as described (Cambier et al., 2014, Elks et al., 2014). After washing in PBST, secondary antibodies conjugated to Alexa Fluors (Molecular Probes) were added at 1:500 and incubated overnight at 4°C.

Bone-marrow derived macrophages (BMDMs) were generated from C57BL/6 mice purchased from The Jackson Laboratory. Bone marrow cells extracted from femora and tibiae of male mice at 6-10 weeks of age were cultured in BMDM media consisting of DMEM (GIBCO) with 20% FBS (Omega), conditioned media containing ∼100 ng/mL M-CSF from L929 cells (kind gift from G. Cheng), and 1X Pen/Strep (GIBCO) for 6 days at 37°C under 4% CO_2_. Cells were washed twice with PBS and medium replaced with antibiotic-free BMDM medium before cells were place in an incubator at 35°C and 4% CO_2_ for at least an hour before infection or stimulation. Cells were infected with *M. leprae* (harvested from footpads of nude mice) at indicated MOI or with equivalent volumes of log-phase (OD_600_ = 0.5 ± 0.1) WT *M. marinum* or *M. marinum*:PGL-1 cultures (growth conditions described below). An equivalent volume of PBS vehicle was added to cell medium for control cells. Approximate MOI for *M. marinum* was calculated from the optical density of the culture, then exact MOI was obtained by growing the cultures on 7H10 plates. MOI for *M. leprae* was calculated based on counting bacilli. For PGL-1 stimulation of cells, PGL-1 purified from the livers of *M. leprae*-infected armadillos (BEI, see Key Resources Table) was resuspended in PBS + 1% DMSO by sonication, then added to cells at a concentration of 10 μg/mL. For control cells, an equivalent volume of PBS + 1% DMSO was added.

Cells were harvested at 0, 2, 6, or 24 hr post-stimulation or infection by addition of 500 μL Trizol (Invitrogen), and RNA extracted using the RNeasy Mini Kit (QIAGEN), as described (Teles et al., 2013). After DNase treatment (QIAGEN) to remove genomic DNA, RNA concentration was obtained by spectrophotometry and equivalent amounts of RNA were used as template for first cDNA strand synthesis, which was performed using the iScript cDNA synthesis kit (BioRad) and a mixture of random hexamer and oligo(dT) primers (Bio-Rad). Real-time PCR of cDNA was performed using SYBR Green (Kapa Biosystems, Roche) fluorescence as a surrogate for transcript abundance; reactions were performed on a CFX96 Realtime System machine (BioRad). To detect fold change in iNOS mRNA abundance, iNOS transcript was normalized to beta actin transcript (see Key Resources Table for primers) and each time-point was compared to control cells using the delta-delta-C_t_ method.

Morpholinos (Gene Tools; see Key Resources Table for sequences) were used to block translation or splicing of transcript for *irf8* (0.6mM) (Li et al., 2011), *pu.1* (mixture of 0.375mM component 1 and 0.025mM component 2) (Clay et al., 2007), *myD88* (5mM) (Bates et al., 2007, Cambier et al., 2014), or *ccr2* (0.3mM) (Cambier et al., 2014). Morpholinos or *in vitr*o-transcribed H2B-CFP (Megason, 2009) were diluted in tango buffer (Thermo Scientific) containing 2% phenol red (Sigma) and injected into the yolk of 1-2 cell-stage embryos in **∼**1 nL (Tobin et al., 2012). Liposomes loaded with clodronate or PBS (van Rooijen et al., 1996) were diluted 1:5 in PBS + 2% phenol red and injected into 2-dpf-old larvae in **∼**10 nL via the caudal vein; liposomes were re-administered every 4 days. To generate larvae with fluorescent mitochondria in axons, eggs were coinjected at the 1-to-4-cell stage with 50 μg/μL *in vitro*-transcribed *tol2* transposase RNA, 25 ng/μL of an existing pDEST-UAS:MLS-dsRed plasmid (O’Donnell et al., 2013), and 25 ng/μL of a constructed pDEST Tol2 plasmid consisting of GAL4 expressed from the *Xenopus laevis* neuronal beta tubulin (*nbt*) promoter (Peri and Nüsslein-Volhard, 2008). To generate larvae with individual labeled oligodendrocytes, eggs were coinjected at the 1-to-4-cell stage with 50 μg/μL *in vitro*-transcribed *tol2* transposase RNA and 1 ng/μL of the mbp:eGFP-CAAX plasmid (Almeida et al., 2011). At 3 dpf, larvae were screened by fluorescence to identify those that had an individual GFP-positive oligodendrocyte near the cloaca; diagrams of these larvae were drawn at 4 dpf to indicate the location of the GFP-positive cell. The diagrams were used to guide injection of bacteria or PBS into the spinal cord adjacent from the cloaca, as closely as possible to the GFP-positive oligodendrocyte. After fluorescence imaging to confirm successful injection, larvae were imaged by confocal (see below).

*M. marinum* M strain (ATCC #BAA-535) and its derivative, *M. marinum:PGL-1*, expressing tdTomato, wasabi or eBFP under control of the msp12 promoter (Cosma et al., 2006, Takaki et al., 2013), were grown under hygromycin (Mediatech) or kanamycin (Sigma) selection in 7H9 Middlebrook medium (Difco) supplemented with oleic acid, albumin, dextrose, and Tween-80 (Sigma) (Takaki et al., 2013). *M. marinum:PGL-1* was constructed by transforming *M. marinum* with the integrating plasmid pWM122, which encodes the *M. leprae* genes ML0126, ML0127, ML0128, ML2346c, ML2347, and ML2348 under the *M. fortuitum pBlaF^∗^* promoter (Tabouret et al., 2010). Kanamycin-resistant transformants were confirmed by PCR using primers targeting all six *M. leprae* genes (Tabouret et al., 2010). A single transformant was further confirmed by mass spectrometry of its phenolic glycolipids; this strain was used for all subsequent experiments. For infections, *M. leprae* was isolated from mouse footpads, labeled with fluorescent dye (PKH67, PKH29, or CellVue Claret, Sigma), then tested for viability by radiorespirometry, as described (Lahiri et al., 2005). Only preparations that exceeded 80% viability were used for infection. Inoculum was calculated based on enumeration performed by the NHDP, with 10^6^ *M. leprae*/μL. *P. aeruginosa* PAO1 expressing GFP has been described (Brannon et al., 2009).

To determine the structure of mycobacterial phenolic glycolipids, *M. marinum* WT and *M. marinum:PGL-1* were cultured in 20 mL of 7H9 medium, supplemented with 10% albumin/dextrose/catalase (EMD Chemicals, San Diego, CA), to mid-log phase (OD_600_ = 0.5 ± 0.1). Total lipids were extracted from cell pellets using 20 mL LC-MS grade chloroform:methanol (Fisher) at 2:1, then 1:1, then 1:2, for 1 hr each, as described (Layre et al., 2011). Collected solvents were dried under nitrogen and total lipids weighed. Each lipid extract, in addition to PGL-1 standard from *M. leprae* (BEI) and PGL-*mar* standard from WT *M. marinum*, was analyzed on an Agilent Technologies 6520 Accurate-Mass Q-Tof and a 1200 series HPLC system with a Varian Monochrom diol column (3 μm x 150 mm x 2 mm) and a Varian Monochrom diol guard column (3 μm x 4.6 mm). Lipids were resuspended at 0.5 μg/mL in solvent A (hexanes:isopropanol, 70:30 [v: v], 0.02% [m/v] formic acid, 0.01% [m/v] ammonium hydroxide), then 10 μg were injected and the column was eluted at 0.15 mL/min with a binary gradient from 0% to 100% solvent B (isopropanol:methanol, 70:30 [v/v], 0.02% [m/v] formic acid, 0.01% [m/v] ammonium hydroxide): 0–10 min, 0% B; 17–22 min, 50% B; 30–35 min, 100% B; 40–44 min, 0% B, followed by additional 6 min 0% B postrun. Ionization was maintained at 325**°**C with a 5 L/min drying gas flow, a 30 psig nebulizer pressure, and 5,500 V. Spectra were collected in positive ion mode from *m/z* 100 to 3,000 at 1 spectrum/s. Continuous infusion calibrants included *m/z* 121.050873 and 922.009798 in positive ion mode. Collision-induced dissociation was performed with an energy of 30 V.

Wide-field microscopy was performed using a Nikon Eclipse Ti-E equipped with a C-HGFIE 130W mercury light source, Chroma FITC (41001) filter, and × 2/0.10 Plan Apochromat objective. Fluorescence images for evaluating bacterial escape from the vasculature were captured with a CoolSNAP HQ2 Monochrome Camera (Photometrics) using NIS-Elements (version 3.22). Quantification of fluorescent bacterial infection, using Fluorescent Pixel Count (FPC) quantification of images of individual embryos, was performed using the FPC macro in ImageJ, as described (Takaki et al., 2012).

For confocal imaging, larvae were imbedded in 1.5% low melting-point agarose (Davis and Ramakrishnan, 2009). A series of z stack images with a 2-3 μm step size was generated through the infected spinal cord with the image centered at the injection site or cloaca, using either the galvo scanner (laser scanner) of the Nikon A1 confocal microscope with a × 20 Plan Apo 0.75 NA objective, or the resonant laser scanner of a Leica TCS-SP5 AOBS confocal microscope with a 20x Plan Apo 0.70 NA. Bacterial burdens were determined by using the three-dimensional surface-rendering feature of Imaris (Bitplane Scientific Software) (Yang et al., 2012). Macrophage numbers, shape and speed were determined using tracking of surface-rendered features on Imaris. When events were compared between larvae, identical confocal laser settings, software settings and Imaris surface-rendering algorithims were used.

Before fixing larvae for TEM, they were imaged by confocal microscopy in order measure the distance from the cloaca to the spinal cord lesion; this allowed sections to be taken through confirmed demyelinating lesions after the larvae were fixed, or through sites of PBS-injection in controls. After rescuing larvae from 1.5% agarose used for confocal imaging, healthy larvae were anesthetized, cooled to 4°C, then fixed overnight in ice-cold 0.1 M sodium cacodylate (Sigma) containing 2% glutaraldehyde (Electron Microscopy Services), 4% paraformaldehyde (Electron Microscopy Services) and 4% sucrose (Fisher) (Czopka and Lyons, 2011). Following several washes in buffer, the larvae were postfixed in a solution of 2% osmium tetroxide (Electron Microscopy Services) and 0.1M imidazole (Electron Microscopy Services) in cacodylate buffer for 1 hr on ice. The larvae were rinsed multiple times in water and treated with 0.5% uranyl acetate (Electron Microscopy Services) overnight at 4°C. They were then dehydrated through a graded series of ethanols (from 30% to 100%), passed through propylene oxide (Electron Microscopy Services) and infiltrated with Eponate12 (Ted Pella) overnight. The larvae were embedded in fresh Eponate12 and the blocks polymerized at 60°C. The areas of interest were identified relative to the cloaca by comparing to confocal imaged taken of the fish before fixation, and 50 nm (silver interference color) sections were taken through these areas on an ultramictome (RMC MTX) and deposited on grids. The grids were stained with saturated uranyl acetate (Electron Microscopy Services) and Reynolds lead citrate (Fisher) and examined on a JEOL 100CX electron microscope at 60kV. Images were collected on film, and then scanned at 1200 dpi to create digital files. Axons were identified by the presence of microtubules and/or microfilaments and an intact outer membrane. Decompacted myelin was identified by the presence of large, electron-lucent spaces in between myelin lamellae that were not observed in the absence of infection. Myelin dissociated from axons was identified by the presence of electron dense “whorls” of myelin lamellae that did not contain an axon. Mitochondria were identified by an intact double membrane and cristae. Axon number, myelination, size, and presence of mitochondria were scored by randomly selecting axons in each image. To assure that axons selection was truly random, each image was opened in its original dimensions in Adobe Photoshop (Adobe, version 12.1) and overlaid with a 50-pixel grid; only axons under grid nodes were scored (Czopka and Lyons, 2011).

### Quantification and Statistical Analysis

Statistical analyses were performed on Prism (version 5.0a, GraphPad). Not significant, p ≥ 0.05; ^∗^ p < 0.05; ^∗∗^ p < 0.01; ^∗∗∗^ p < 0.001; ^∗∗∗∗^ p ≤ 0.0001.

### Data and Software Availability

The following software was used: Adobe Photoshop and Adobe Illustrator (quantification of axons, myelin and mitochondria in TEMs; figure preparation), ImageJ (quantification of axons, myelin and mitochondria in TEMs; bacterial burden by FPC), and Imaris (tracking and rendering confocal objects); see Key Resources Table for more information.

## Author Contributions

C.A.M. and L.R. conceived and designed all experiments, except those represented in Figures 1C and 1D (C.J.C. and L.R.); Figures 3F and 7A and Movie S2 (A.S.); Figure S2 (C.A.M., T.-Y.C., and D.B.M.); and Figure S5A (C.A.M., P.O.S., and S.T.S.). C.A.M. performed all the experiments, except those represented in Figures 1C and 1D (C.J.C.), Figure S2 (T.-Y.C.), and Figure S5A (J.Z., K.M.K.-S., and P.O.S.). C.A.M. and L.R. analyzed all data, except for those represented in Figures 1C and 1D (C.J.C. and L.R.) and Figure S5A (J.Z., K.M.K.-S., and P.O.S.). C.A.M. and L.R. designed all the figures. C.A.M. prepared all the figures, except for Figure S2 (T.-Y.C.). C.A.M. and L.R. wrote the paper with input from A.S. T.-Y.C. and D.B.M. provided biochemical analysis of glycolipids made by mutant strains and contributed to writing the manuscript. B.R.B. and R.L.M. contributed to the experimental design, interpretation, and writing. L.R. conceived the idea to use the zebrafish to study leprosy and oversaw the project.

## Supplemental Information

Document S1. Table S1

Movie S1. Rotating Rendering of a Demyelinating Nerve Lesion, Related to Figures 2F and 2G An *mbp:eGFP-CAAX* larva (green myelin) at 3 days post-fertilization was injected in the spinal cord with *M. leprae* (red), then imaged by confocal microscopy at 4 days post-infection. 10μm bar.

Movie S2. Time-Lapse Imaging of Myelin Protrusions Forming from Individual Oligodendrocytes, Related to Figure 3 Time-lapse movies of individual oligodendrocytes in the spinal cord of transient *mbp:eGFP-CAAX* transgenic larvae. Larvae were injected in the spinal cord near the labeled cell (see the STAR Methods) and imaged immediately for 12 hours. Injection sites are out of the frame to the left. 10μm bar; relative timecode. (A) Injection of *M. marium*:*PGL-1.* Beginning ∼5 hours post-injection, the apparently intact myelin sheath bulges and 3 protrusions begin to form (arrows). (B) Injection as in (A) of PBS vehicle.

Movie S3. Time-Lapse Confocal Imaging of Macrophages in the Spinal Cord of Uninjected Larvae, Related to Figure 5 An *mbp:eGFP-CAAX;mpeg1:Brainbow* larva (green myelin, red macrophages), at 4 days post-fertilization, imaged in the spinal cord adjacent to the cloaca. Myelin is shown as the fluorescence image and macrophages are shown as renderings. There is green autofluorescence from the skin dorsal to the myelinated axons, which covers the dorsal aorta. The autofluorescence is indicated by a rendered blue overlay. An arrow indicates a macrophage that associates with the myelin. Only a portion of the 12-hour movie is shown. 10μm bar; relative timecode.

Movie S4. Time-Lapse Confocal Imaging of Macrophages in the Spinal Cord of Uninjected Larvae, Related to Figure 5 Another example of an untouched larvae, imaged and displayed as in Movie S3. An arrow indicates a macrophage that associates with the myelin.

Movie S5. Time-Lapse Confocal Imaging of Macrophages in the Spinal Cord of PBS-Injected Larvae, Related to Figure 5 An *mbp:eGFP;mpeg1:Brainbow* larva (green myelin, red macrophages), at 4 days post-fertilization, was injected in the spinal cord with PBS + 2% phenol red and immediately imaged by confocal microscopy. Myelin is shown as the fluorescence image and macrophages are shown as renderings. Macrophages initially cluster at the injection site (circle) and crawl along myelinated axons (arrows); however, few or no macrophages remain associated with myelin after ∼8 hours post-injection. 10μm bar; relative timecode.

Movie S6. Time-Lapse Imaging of *M. leprae* Injection in the Spinal Cord, Related to Figure 5C An *mbp:eGFP-CAAX;mpeg1:Brainbow* larva (green myelin, red macrophages) at 4 days post-fertilization was injected in the spinal cord with *M. leprae* (cyan), imaged and displayed as in Movie S3. At the injection site (circle), a small aggregate of heavily-infected macrophages remain for the duration of imaging. Meanwhile, uninfected and infected macrophages, with bacilli visible inside of them, arrive at the injection site by crawling along or pushing apart myelinated axons (arrows). After phagocytosing bacilli or contacting other cells, infected macrophages crawl away from the injection site, carrying *M. leprae*. Myelin is shown as the fluorescence image; macrophages are shown as transparent renderings to show the rendered *M. leprae* inside. The imaged area, centered around the injection site, has dimensions x = 200μm, y = 95μm, z = 50μm. 10μm bar; relative timecode.

Movie S7. Time-Lapse Imaging of *M. marinum* Injection in the Spinal Cord, Related to Figures 5D–5F An *mbp:eGFP-CAAX;mpeg1:Brainbow* larva (green myelin, red macrophages cells), at 4 days post-fertilization, was injected with ∼100 colony-forming units of *M. marinum* (cyan), imaged and displayed as in Movie S3. The macrophage response is similar to that in *M. leprae* infection, with several heavily-infected macrophages remaining at the injection site (circle) for most of imaging. Macrophages crawl along the myelin sheaths (arrows) toward the injection site, phagocytose bacilli, and exit the frame. Macrophage morphology, speed, and myelin colocalization are quantified in Figures 5D–5F.

Movie S8. Time-Lapse Imaging of *M. marinum:PGL-1* Injection in the Spinal Cord, Related to Figures 5D–5F An *mbp:eGFP-CAAX;mpeg1:Brainbow* larva (green myelin, red macrophages), at 4 days post-fertilization, was injected with ∼100 colony-forming units of *M. marinum*:PGL-1 (cyan), imaged and displayed as in Movie S3. As with *M. leprae* and *M. marinum* injection, several heavily-infected macrophages remain at the injection site (circle). Macrophages crawl along the myelin sheaths (arrows) toward the injection site, phagocytose bacilli, and exit the frame for distal sites. Macrophage morphology, speed, and myelin colocalization appear similar to that in *M. marinum* infection (quantified in Figures 5D–5F).
