# Supplementary material for: A Macrophage Response to Mycobacterium leprae Phenolic Glycolipid Initiates Nerve Damage in Leprosy
Source: Cell. 2017 Aug 24;170(5):973–985.e10. doi: 10.1016/j.cell.2017.07.030 (PMC5848073; doi:10.1016/j.cell.2017.07.030)
Supplement: Document S1. Table S1 [file mmc1.pdf]

Cell, Volume *170*

Supplemental Information

A Macrophage Response to *Mycobacterium leprae* Phenolic Glycolipid Initiates Nerve Damage in Leprosy

Cressida A. Madigan, C.J. Cambier, Kindra M. Kelly-Scumpia, Philip O. Scumpia, Tan-Yun Cheng, Joseph Zailaa, Barry R. Bloom, D. Branch Moody, Stephen T. Smale, Alvaro Sagasti, Robert L. Modlin, and Lalita Ramakrishnan

Table S1 Expression of *M. leprae*-determinants in Schwann cells and oligodendrocytes, with their respective functions. Related to Figure 2.

marker	interaction with M. leprae	Schwann cell function	oligodendrocyte function	references
myelin basic protein	demyelination in patients	myelin constituent	myelin constituent	Scollard 2006
ErbB2	Mlep signalling via ErbB2 promotes proliferation	promotes differentiation, myelination, proliferation	promotes differentiation	Tapinos 2006; Park 2001
Laminin 2, α-dystroglycan	Lep ML-LBP-21 and PGL1 bind, promoting internalization	attches SC to ECM	attaches oligos to ECM	Shimoji 1999; Ng 2000; Rambukkana 1998; Leiton 2015

Adapted from Scollard 2008, Table I (Scollard, 2008)
